# Lysophosphatidic acid suppresses apoptosis of high-grade serous ovarian cancer cells by inducing autophagy activity and promotes cell-cycle progression *via* EGFR-PI3K/Aurora-A^Thr288^-geminin dual signaling pathways

**DOI:** 10.3389/fphar.2022.1046269

**Published:** 2022-12-19

**Authors:** Haile Zhao, Peijun Jia, Kathleen Nanding, Man Wu, Xiaozhou Bai, Morigen Morigen, Lifei Fan

**Affiliations:** Inner Mongolia Key Laboratory for Molecular Regulation of the Cell, State Key Laboratory of Reproductive Regulation and Breeding of Grassland Livestock, School of Life Sciences, Inner Mongolia University, Hohhot, China

**Keywords:** LPA, geminin, EGFR transactivation, autophagy, apoptosis, Aurora-A^Thr288^

## Abstract

Lysophosphatidic acid (LPA) and geminin are overexpressed in ovarian cancer, and increasing evidence supports their contribution to ovarian tumor development. Here, we reveal that geminin depletion induces autophagy suppression and enhances reactive oxygen species (ROS) production and apoptosis of high-grade serous ovarian cancer (HGSOC) cells. Bioinformatics analysis and pharmacological inhibition studies confirm that LPA activates geminin expression in the early S phase in HGSOC cells *via* the LPAR_1/3_/MMPs/EGFR/PI3K/mTOR pathway. Furthermore, LPA phosphorylates Aurora-A kinase on Thr288 through EGFR transactivation, and this event potentiates additional geminin stabilization. In turn, overexpressed and stabilized geminin regulates DNA replication, cell-cycle progression, and cell proliferation of HGSOC cells. Our data provide potential targets for enhancing the clinical benefit of HGSOC precision medicine.

## Introduction

Ovarian cancer is a frequently lethal malignancy affecting the female reproductive tract. Histologically, about 90% of ovarian tumors are considered to occur through the transformation of epithelial cells (Matulonis et al., 2016). Epithelial ovarian cancer (EOC) typically includes at least four major histological subtypes: serous, endometrioid, clear cell, and mucinous carcinoma (Seidman et al., 2004). The most aggressive serous subtype, high-grade serous ovarian cancer (HGSOC), accounts for 90% of these serous carcinomas and 70%–80% of all ovarian cancer deaths (Bowtell, 2010; Mitra et al., 2015). In fact, HGSOC predominates in the clinical setting, making it the most extensively studied ovarian cancer (Lisio et al., 2019).

HGSOC is characterized by the rapid growth and spread of intraperitoneal tumors and the accumulation of ascites. HGSOC patients with late-stage disease frequently develop malignant ascites featuring a prominent cellular component (Lengyel, 2010). Lysophosphatidic acid (LPA) is a bioactive phospholipid that performs an important signal messenger role in both physiological and pathological conditions. The level of LPA in plasma or ascites is obviously elevated in most ovarian cancer patients with poor prognostic outcomes, and LPA is considered a potential diagnostic biomarker for ovarian cancer (Xu et al., 1995; Xu et al., 1998; Baker et al., 2002; Mills and Moolenaar, 2003; Jesionowska et al., 2015). Enhanced LPA connotes rapid and constant cell growth, typically accompanied with replication stress, which is defined as aberrant replication fork progression, DNA synthesis, and uninterrupted generation of chromosome variations (Zeman and Cimprich, 2014). Ultimately, these abnormal variations promote oncogenic transformation (Zeman and Cimprich, 2014; Kotsantis et al., 2018; Maya-Mendoza et al., 2018).

Cancer cells can recruit and change the expression of key regulatory factors, especially oncogenes, to modulate cancer progression. Aurora-A is over-expressed in ovarian cancers and shows a contribution to ovarian tumor development (Yang et al., 2004; Chung et al., 2005; Jr et al., 2007). Aurora-A kinase induces telomerase activity and human telomerase reverse transcriptase (hTERT) by upregulation of c-Myc (Yang et al., 2004), regulates RNA metabolism (Adhikari et al., 2020). Activated Aurora-A can suppress R-loop formation and transcription-replication conflicts by stabilizing MYCN (Roeschert et al., 2021) and enhance the oncogenic RNA splicing of tumor suppressor RBM4 by switching m6A reader YTHDC1 (Li et al., 2022). Aurora-A induces cancer cell division and growth by mediating pre-metaphase events, such as centrosome biology and bipolar spindle assembly (Marumoto et al., 2005; Jr et al., 2007; Barretta et al., 2016; Gallini et al., 2016; Zhao et al., 2019; Lai et al., 2020). Subsequently, Aurora-A is confirmed to phosphorylate geminin on Thr25 and then prevent geminin degradation in mitosis (Tsunematsu et al., 2013). As both a negative and positive regulator of the pre-replication complex (pre-RC), geminin directly binds to chromatin licensing and DNA replication factor 1 (Cdt1) to regulate nuclear DNA initiation (Wohlschlegel James et al., 2000; Ballabeni et al., 2004; Lee et al., 2004). Aberrant expression or degradation of geminin ultimately triggers genomic instability and oncogenic transformation (Zhu & Depamphilis, 2009; Champeris Tsaniras et al., 2018; Ma et al., 2021). Oncogenic transformation contributes to reactive oxygen species (ROS) production (Park et al., 2014; Maya-Mendoza et al., 2015), which in turn influences the occurrence of replication stress through polymerase activity or the physical obstacle of oxidizing dNTPs and dissociation of peroxiredoxin2 oligomers (PRDX2) (Sedletska et al., 2013; Park et al., 2014; Maya-Mendoza et al., 2015; Somyajit et al., 2017; Meng et al., 2018). ROS production has also been reported to induce cell apoptosis of many cancers (Grishko et al., 2009; Kim et al., 2010). Autophagy serves as a dynamic recycling system of eukaryotic cells that drives cellular renovation and maintains cellular homeostasis (Mizushima and Komatsu, 2011). Recent studies have proven that autophagy protects cells from DNA damage, genomic instability, and apoptosis by reducing ROS production (Mathew et al., 2007; Gibson, 2013; Shen et al., 2015). However, the molecular–genetic mechanisms of HGSOC remain obscure, and it would be meaningful to analyze the relationship between geminin, ROS, and autophagy in HGSOC cancers. Furthermore, the investigation of critical proteins’ biological functions has been based on traditional two-dimensional (2D) monolayer cultures. An *in vitro* three-dimensional (3D) multicellular spheroid model is better for screening and target identification.

In this study, we linked geminin levels to ROS production, autophagy, and apoptosis in HGSOC cells. Depletion of geminin suppressed autophagy, enhanced ROS production, and induced apoptosis. In addition, the rapid and constant cell growth induced by LPA was convincingly highlighted in long-term 3D cultures. We further investigated the signaling and physiological function of regulatory factors or oncogenes upon LPA stimulation in HGSOC cells.

## Materials and methods

### Two-dimensional cell culture

A2780 cells were maintained in complete growth medium containing DMEM (high glucose, Gibco, MD, United States) supplemented with 10% fetal bovine serum (DCELL biologics, Shanghai, China), 1% penicillin-streptomycin (Gibco), and 1% L-glutamine (Gibco). OVCAR5 cells were maintained in complete growth medium containing RPMI 1640 (Gibco) supplemented with 10% fetal bovine serum (DCELL biologics), 1% penicillin-streptomycin (Gibco), and 1% L-glutamine (Gibco). All cells were cultured at 37 °C with 5% CO_2_ in a humidified atmosphere (Thermo Fisher Scientific, MA, United States). In a series of experiments in this article, cells were synchronized in the G1/S-phase with a serum-free medium for 24 h and then treated with specified reagents (LPA, EGF, DMSO, inhibitors) in a serum-free medium supplemented with 1% BSA (Yarwood and Woodgett, 2001; Hu et al., 2009; Bousette et al., 2010).

### Three-dimensional cell culture

A2780 cells and OVCAR5 cells were cultured in CERO cell culture simplified (OMNI Life Science, Bremen, Germany); 1×10^6^ cells were inoculated for each Falcon tube. During the cultivation, cells were grown in suspension culture with the following setup: Inoculation period: rotation pause, 0 s; rotation period, 1 s; agitation pause, 40 min. Agitation period: 2 min; rotation speed, 50 rpm; duration, 4 h. The culture period (protocol) was the following for 8 or 16 days: rotation pause, 0 s; rotation period, 1 s; agitation pause, 0 s. Agitation period: 2 min; rotation speed, 80 rpm; Duration, ∞. Falcon tubes were filled with 40 ml complete growth medium. The culture medium was changed once after 72 h by removing 30 ml medium and replacing it with the complete growth medium depending on the cell type.

### Reagents and antibodies

Reagents were from the following suppliers: LPA (L7260), DMSO (D2650), BSA (A6003), propidium iodide (PI, P4170) (Sigma, UK); human-EGF (PHG0313; Gibco); Ki16425 (S1315), batimastat (BB94, S1715), AG1478 (S2728), LY294002 (S1105), rapamycin (S1039), CCT137690 (S2744) (Selleck); 4’,6-diamidino-2-phenylindole (DAPI, D3571) (Invitrogen, Carlsbad, CA, United States). Antibodies were from the following suppliers: anti-phosphotyrosine (EPR16871; Abcam); anti-GAPDH (HC301), anti-β-actin (HC201), anti-β-tubulin (HC101), anti-rabbit (HS101), and anti-mouse (HS201) antibodies (TransGen Biotech, Beijing, China); anti-Bax (bs0127R), anti-Bcl 2 (bs0032R) antibodies (Bioss, Beijing, China); anti-BrdU (abs128684), anti-AURKA (abs136365), anti-phospho-Aurora kinase (Thr288) (abs130639) antibodies (Absin, Shanghai, China); anti-caspase 3/p17 (19677-1-AP), anti-caspase 6/p18/p11 (10198-1-AP), anti-caspase7 (27155-1-AP), anti-caspase 9 (10380-1-AP), anti-PARP (13371-1-AP), anti-EGFR (18986-1-AP), anti-geminin (10802-1-AP) antibodies (Proteintech Group, Wuhan, Hubei, China); anti-LC3B (AF4650, Affinity Biosciences, United States); Alexa Fluor 488–conjugated goat anti-mouse IgG (H + L) antibody (A11008, Invitrogen).

### RNA interference and transfection

To knock down endogenous geminin expression, 1 × 10^6^ A2780 cells were transfected with 15 μg shRNA plasmid (Sangon Biotech, Shanghai, China) that targeted human *GMNN* gene using Lipofectamine 2000 (11668-027; Thermo Fisher Scientific) according to the manufacturer’s instructions. The knockdown efficiency of endogenous geminin expression was confirmed by immunoblotting assay. The shRNA (*GMNN*-homo-315) and shNC (negative control) sequences are described in Supplementary Table S1.

### Immunoblotting and immunoprecipitation

Cells were washed three times with 1× phosphate-buffered saline (PBS). Total protein was extracted with 1× lysis buffer. Protein concentrations were measured using a Pierce™ BCA Protein Assay Kit (23227; Thermo Fisher Scientific). Proteins were separated by SDS-PAGE and electro-transferred to a semi-dry PVDF membrane (Bio-Rad, Hercules, CA, United States). Membranes were blocked in PBST containing 5% non-fat milk (BD Biosciences, San Jose, CA, United States) for 1 h at room temperature, incubated with specified primary overnight at 4 °C and secondary antibodies for 1 h at room temperature. The protein bands were visualized using Pierce ECL Western Blotting Substrate (32106; Thermo Fisher Scientific). The relative amounts of proteins were quantified by densitometry using the ChemiDoc XRS system (Bio-Rad) and calculated using Image Lab Version 5.2.1 (Bio-Rad) for each time point. For immunodetection, the dilution ratio was 1:1,000 for all primary antibodies and 1:20,000 for all secondary antibodies, respectively. For the immunoprecipitation (IP) assay, the lysate containing 4–5 mg total protein is applied for one assay. An aliquot of 4 μg of the primary antibody was added to the lysate, and the mixture was gently rocked at 4.0 °C for 12 h followed by incubation with 50 μL Protein G Sepharose beads slurry (P3296; sigma) to capture the immunocomplex. After another 12 h incubation at 4.0 °C, the mixture was washed three times with pre-cooled 1× lysis buffer, and the supernatant were discarded. The pellet was resuspended with 20 μL 5× SDS sample buffer. Proteins were then detected by immunoblotting assay (Zhao et al., 2021).

### Apoptosis analysis

Apoptosis was measured by flow cytometry (FCM) using an FITC Annexin V Apoptosis Detection Kit I (556547; BD Biosciences) (Bhutia et al., 2010). When the cells had grown to 80% confluence, A2780 cells were transfected with 15 μg shNC or sh-*GMNN* plasmid for 6 h and cultured for another 48 h in complete growth medium until harvested. The manufacturer’s instructions were followed to collect and analyze 1×10^5^ cells. FCM analysis was performed using a CytoFLEX Flow Cytometer (Beckman Coulter Life Sciences, United States), and data were analyzed using CytExpert (Beckman Coulter) software.

### Intracellular reactive oxygen species (ROS) detection

Intracellular ROS levels were evaluated using a Reactive Oxygen Species Assay Kit (CA1410; Solarbio Science and Technology, Beijing, China) according to the manufacturer’s instructions. When the cells had grown to 80% confluence, A2780 cells were transfected with 15 μg shNC or sh-*GMNN* plasmid for 6 h in OptiMEM medium and cultured for another 48 h in complete growth medium until harvested. The analysis was performed using the CytoFLEX Flow Cytometer (Beckman Coulter Life Sciences), and data were analyzed using CytExpert (Beckman Coulter) software.

### Quantitative real-time PCR

Total RNA was extracted from cells using TRIzol (ET111; TransGen Biotech) according to the manufacturer’s instructions. A HiScript II 1st Strand cDNA Synthesis Kit (+gDNA wiper) (Vazyme Biotech) was used to generate the cDNA template. qRT-PCR was performed with TransStart Tip Green qPCR SuperMix (TransGen Biotech) on a LightCycler 480 II system (Roche, Basel, Switzerland) (Zhao et al., 2021). Oligonucleotide sequences (Sangon Biotech) are described in Table S2.

### BrdU staining and immunofluorescence analysis

Cells were seeded on glass coverslips in a 12-well plate and allowed to adhere overnight. After synchronization with a serum-free medium for 24 h, OVCAR5 cells were treated with 0.1% DMSO (vehicle), 10 μM LPA or 10 ng/ml EGF for 4 h, and incubated with 10 μM BrdU labeling solution (in water) for 24 h. The BrdU labeling solution was removed, and cells were washed five times with PBS. OVCAR5 cells were fixed in 4% paraformaldehyde for 15 min, permeabilized with Triton X-100 for 5 min, incubated in sodium borohydride for 10 min, stained with mouse anti-BrdU antibody (1:200 in 1% BSA) for 1 h, and then fluorescein Alexa Fluor 488–conjugated goat anti-mouse IgG (H + L) secondary antibody (1:200 in 1% BSA) for 45 min at room temperature. DNA was visualized by staining with DAPI (1:5,000 in water). The immunofluorescence analysis was visualized with a laser scanning confocal microscope (LSM 710, CarlZeiss, Wetzlar, Germany) (Zhao et al., 2021).

### Cell proliferation analysis

Cells (2,000) were seeded on a 96-well plate with complete growth medium and allowed to adhere overnight. After synchronization with a serum-free medium for 24 h, OVCAR5 cells were pretreated with DMSO (vehicle, 0.1%), Ki16425 (10 μM), BB94 (10 μM) or AG1478 (250 nM) for 30 min, and then grown in 0.1% DMSO, 10 μM LPA or 10 ng/ml EGF. The assay was conducted using a Cell Counting Kit-8 (BS360A; Biosharp life sciences, Anhui, China) according to the manufacturer’s protocol. The plate was then read using a spectrophotometric microtiter plate reader (EPOCH) set at a wavelength of 450 nm.

### DNA ploidy analysis

OVCAR5 cells were synchronized with a serum-free medium for 24 h and then pretreated with DMSO (vehicle, 0.1%), Ki16425 (10 μM), BB94 (10 μM), AG1478 (250 nM), LY294002 (10 μM) or rapamycin (100 nM) for 30 min, stimulated with 10 μM LPA up to 4 h. Cell-cycle progression under different conditions was evaluated by flow cytometry. After the treatment, cells were harvested by digestion with 0.05% trypsin, washed three times with ice-cold 1×PBS, fixed with ice-cold 70% ethanol for 30 min on ice, and incubated with 50 μg/ml PI and 50 μg/ml RNase A in 1×PBS for 30 min in the dark. All FACS analyses were performed on a FACSCalibur system (BD Biosciences), and cell cycle distribution was analyzed with ModFit LT v3.0 software (Verity Software House, Topsham, ME, United States).

### Data source

Human ovarian cancer tissues, normal ovarian tissues, and clinical and molecular data (including mRNA expression and mutations) were extracted from the Cancer Genome Atlas (TCGA) (Grossman et al., 2016). The immunohistochemistry (IHC) data were extracted from the Human Protein Atlas (HPA) (Uhlén et al., 2005). The Kaplan–Meier survival analysis data was extracted from the Kaplan–Meier plotter (Győrffy, 2021). The protein–protein interaction (PPI) network was extracted from STRING (Szklarczyk et al., 2021). The correlation analysis was extracted from GEPIA (Tang et al., 2017), and the Spearman method was used to calculate the correlation coefficient. The gene set enrichment analysis (GSEA) was extracted from the LinkedOmics Database (Vasaikar et al., 2018), the platform is HiSeq RNA, and the pipeline is Firehose_RSEM_log2. The signaling pathway was drawn from Figdraw (www.figdraw.com), and we have the corresponding export copyright ID code.

### Statistical analysis

Statistical information for individual experiments can be found in the corresponding figure legends. All statistical analyses were processed using GraphPad Prism v8.0 software or OriginPro 2021 software. Statistical comparisons were analyzed for significance by Student’s unpaired *t*-test, one-way or two-way analysis of variance (VNOVA) with Dunnett’s multiple comparison test. *p < 0.05, **p < 0.01, and ***p < 0.001 were considered to be statistically significant. Data are presented as mean ± SEM.

## Results

### Geminin expression levels are increased but strictly conservative in human ovarian cancer tissues

From the cytological perspective, HGSOC cells are characterized by high-grade nuclear atypia with large, hyperchromatic, and pleomorphic nuclei with multinucleation potential (Lisio et al., 2019). The public database was interrogated to investigate why disruption of the nuclear atypia would benefit a cancer cell. The *GMNN* transcription level was clearly higher in most human cancer tissues than that in normal tissues (Supplementary Figure S1A). Moreover, human ovarian cancer tissues exhibited significantly increased *GMNN* transcript levels compared with normal ovarian tissues (*p* < 0.001) (Supplementary Figure S1B). In the clinicopathologic characteristics, the *GMNN* transcript level was significantly different among the different pathological stages of ovarian cancer (*p* < 0.05) (Supplementary Figure S1C), but this data set did not show a significant correlation between *GMNN* expression and patient age distribution (Supplementary Figure S1D). Further IHC analysis revealed that geminin protein expression was notably detectable in ovarian cancer tissues, whereas a weak signal was detected in normal ovarian tissues (Supplementary Figure S1E). In addition, data on TCGA whole-genome sequencing cohort revealed only three mutations (H57Q, W99C, E123E) in 165 ovarian cancer patients, suggesting that the geminin protein was strictly conserved (Supplementary Table S3). In brief, these data suggest that geminin expression levels in human ovarian cancer tissues are higher than that in normal ovarian tissues.

### Depletion of geminin suppresses autophagy activity, enhances ROS production, and induces apoptosis of HGSOC cells

**FIGURE 1:**
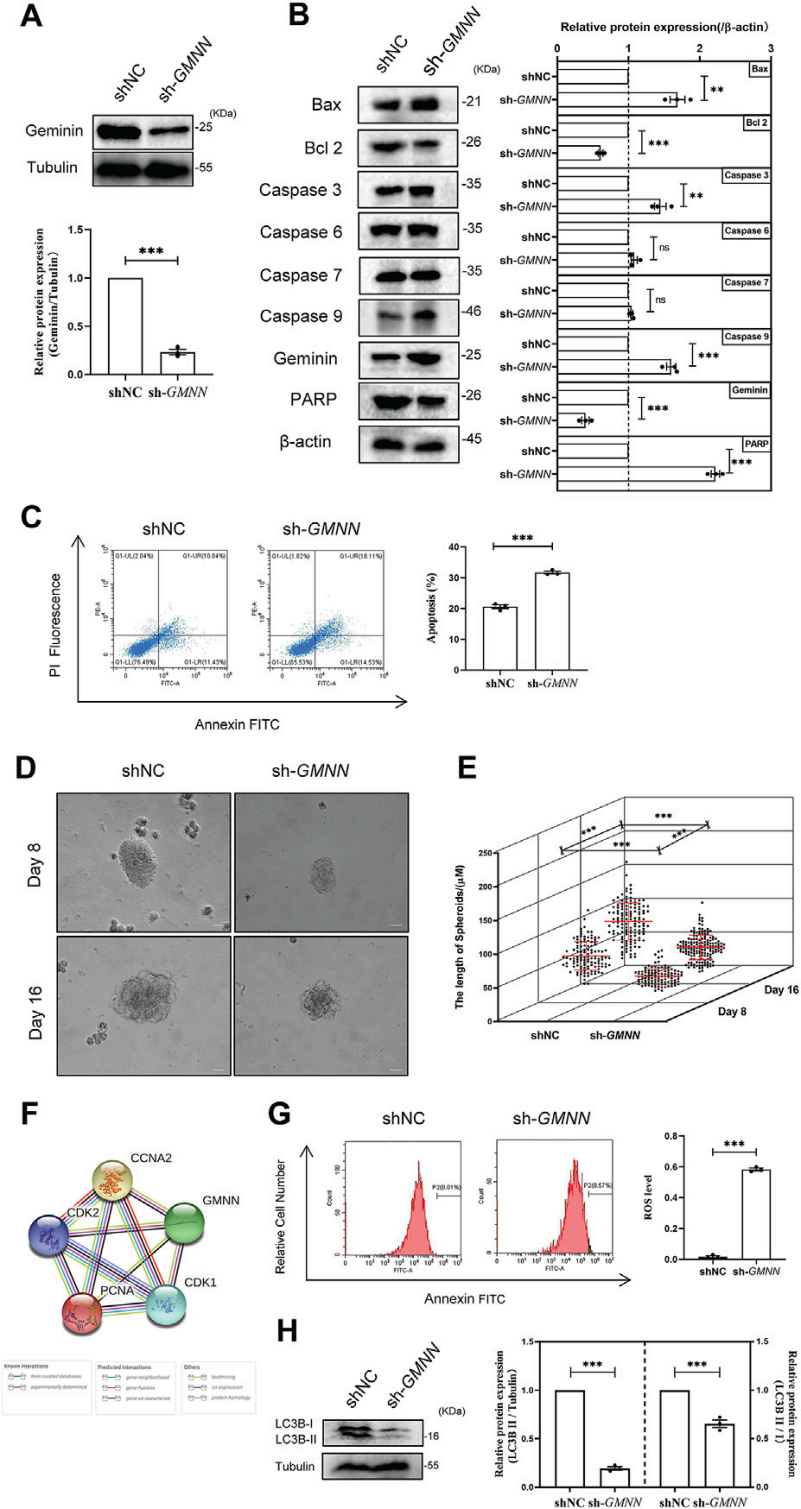
Depletion of geminin suppresses autophagy activity, enhances ROS production, and induces the apoptosis of HGSOC cells. **(A)** Immunoblotting analysis of geminin protein levels in A2780 cells as indicated. shNC, A2780 cells transfected with shNC; sh-*GMNN*, geminin-knockout A2780 cells transfected with shRNA. Quantification of three independent experiments was performed, normalized to tubulin, and expressed as a ratio of NC, mean ± SEM, Student’s unpaired *t*-test, ***p < 0.001. **(B)** IB analysis of apoptosis-related proteins’ expression levels in A2780 cells as indicated. Mean ± SEM, *n* = 3, Student’s unpaired *t*-test, **p < 0.01, ***p < 0.001, ns—non-significant. **(C)** Apoptosis analysis of A2780 cells under geminin depletion. The percentage of apoptotic cells was analyzed with flow cytometry. Mean ± SEM, *n* = 3, Student’s unpaired *t*-test, ***p < 0.001. **(D)** Representative phase-contrast images of bioreactor expanded A2780 cells at indicated days of cultivation. Scale bar 20 μm. **(E)** Graphical illustration of the average aggregate size measured at the indicated day of cultivation: Day 8, shNC *n* = 116, sh-*GMNN* *n* = 108; Day 16, shNC *n* = 130, sh-*GMNN* *n* = 161. Images were analyzed using Nikon NIS Elements D software, mean ± SD, Student’s unpaired *t*-test, ***p < 0.001. **(F)** The protein–protein associations about geminin protein. Data from STRING. **(G)** ROS analysis of A2780 cells under geminin depletion. The percentage of apoptosis cells was analyzed with flow cytometry. Mean ± SEM, *n* = 3, Student’s unpaired *t*-test, ***p < 0.001. **(H)** IB analysis of LC3B protein levels in A2780 cells as indicated. Mean ± SEM, *n* = 3, Student’s unpaired *t*-test, ***p < 0.001.

To define the impact of geminin on human ovarian cancer initiation and progression, geminin-depleted HGSOC cells were established by treating A2780 cells using shRNA to knock out endogenous geminin (Figure 1A, Supplementary Table S1). The Bcl-2 family proteins are key regulators of mitochondrial apoptosis and cysteine-aspartic proteases (caspases) are used as cell death markers (Green and Kroemer, 2004; Kale et al., 2018; Singh et al., 2019). The results of immunoblotting manifested a significant decrease in anti-apoptotic protein Bcl-2 and a significant increase in pro-apoptotic protein Bax, showing geminin depletion resulted in the promotion of cell apoptosis (Figure 1B). In addition, this change in the dynamic balance of two proteins led to markedly increased caspase 9 levels (Figure 1B). An obvious increase was also observed in caspase 3 upon depletion of geminin, while no changes were seen in caspase 6 and caspase 7. These results suggest that the downstream cascade of events is caspase 3 independent (Figure 1B). The FCM analysis showed that apoptotic cells increased from 21.47% to 32.64% towards geminin depletion, indicating that genetic ablation of geminin induced the apoptosis of A2780 cells (Figure 1C). The further 3D assay showed an over-1.7-fold increase in the size of tumor spheroids in geminin knockdown cells compared to the control group (Figures 1D, E). Furthermore, the PPI network revealed that geminin was implicated in the cellular response to ROS with other four proteins (PCNA, CDK1, CDK2, CCNA2) (Figure 1F), and the fraction of cells with intracellular ROS production increased 19 to 60-fold within 2 days with geminin depletion (Figure 1G).

Recent studies demonstrated that autophagy could protect cells by reducing ROS production (Mathew et al., 2007). The expression of autophagy-related protein LC3B was detected to identify whether geminin might affect autophagy activity in HGSOC cells. The results of immunoblotting showed that LC3B protein levels significantly decreased in geminin-depleted A2780 cells (Figure 1H). This finding suggests that geminin protein levels are essential for LC3B protein expression and autophagy activity in HGSOC cells. Overall, these findings imply that geminin depletion suppresses autophagy activity, enhances ROS production, and ultimately induces apoptosis in HGSOC cells.

### LPA activates geminin expression in HGSOC cells

**FIGURE 2:**
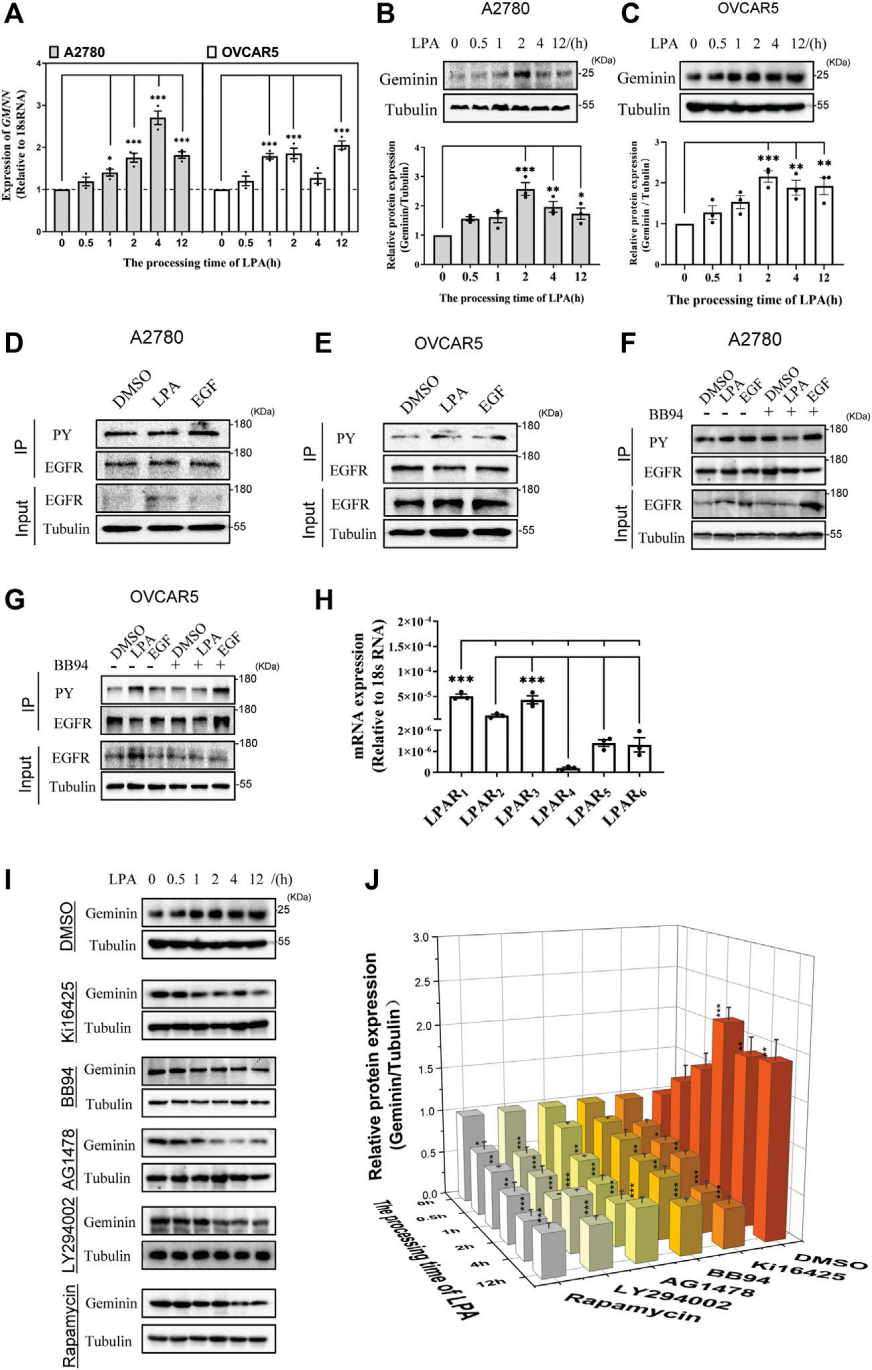
LPA enhances geminin expression *via* a LPAR_1/3_/MMPs/EGFR/PI3K/mTOR signalling pathway in HGSOC cells. **(A)** The mRNA expression levels of geminin in A2780 cells and OVCAR5 cells. VNOVA, Dunnett’s Multiple Comparison Test, mean ± SEM, *n* = 3, *p < 0.05, ***p < 0.001. **(B,C)** Immunoblotting analysis of geminin protein levels in A2780 cells **(B)** and OVCAR5 cells **(C)** under a 10 μM LPA time gradient stimulus. Quantification of three independent experiments was performed, normalized to tubulin, and expressed as a ratio of 0 h, respectively. Mean ± SEM, VNOVA, Dunnett’s Multiple Comparison Test, *p < 0.05, **p < 0.01, ***p < 0.001. **(D,E)** Immunoprecipitation analysis with tyrosine-phosphorylated EGFR antibodies (PY) in A2780 cells **(D)** and OVCAR5 cells **(E)** treated with 0.1% DMSO (vehicle), 10 μM LPA or 10 ng/mL EGF for 5 min. **(F,G)** IP analysis with tyrosine-phosphorylated EGFR antibodies (PY) in A2780 cells **(F)** and OVCAR5 cells **(G)** pretreated with or without 10 μM BB94 for 30 min, and then stimulated with 0.1% DMSO (vehicle), 10 μM LPA or 10 ng/mL EGF for 5 min. **(H)** The mRNA expression of LPARs in OVCAR5 cells. Mean ± SEM, *n* = 3, VNOVA, Dunnett’s Multiple Comparison Test, ***p < 0.001. **(I)** IB analysis of geminin protein levels in OVCAR5 cells as indicated. Cells were pretreated with 0.1% DMSO (vehicle), 10 μM Ki16425, 10 μM BB94, 10 μM AG1478, 10 μM LY294002 or 100 nM Rapamycin for 30 min, and then stimulated with 10 μM LPA time gradient. **(J)** Quantificantion of IB in **(I)**. Data are analyzed with OriginPro 2021, mean ± SEM, *n* = 3, VNOVA, Dunnett’s Multiple Comparison Test, *p < 0.05, **p < 0.01, ***p < 0.001.

HGSOC cells were reported to produce LPA in the peritoneal tumor microenvironment (Ghoneum et al., 2018). To verify the effect of LPA on geminin, qRT-PCR analysis was performed to examine geminin expression in HGSOC cell lines (A2780 and OVCAR5) treated with LPA. The data revealed that the *GMNN* transcript levels were enhanced by LPA stimulus in a time-dependent manner, suggesting that LPA can induce geminin synthesis (Figure 2A). An immunoblotting assay was performed to examine the levels of geminin protein towards the LPA treatment. Consistently, LPA stimulus led to increased geminin protein in a time-dependent manner (Figures 2B, C). Thus, LPA can activate geminin protein expression in HGSOC cells.

### Matrix metalloproteinases play a pivotal role in LPA-induced EGFR transactivation

LPA was clarified to transactivate the epidermal growth factor receptor (EGFR) in a classic autocrine manner in some cancer cells (Daub et al., 1996; Prenzel et al., 1999). In HGSOC cells, the analysis of immunoprecipitation showed that phosphorylated EGFR protein increased notably in the presence of LPA (Figures 2D, E), indicating that LPA enhances EGFR protein phosphorylation. To further examine how LPA transactivates EGFR, HGSOC cells were treated with BB94 to abolish the activity of matrix metalloproteinases (MMPs) responsible for EGFR transactivation. The analysis showed that the increasing phosphorylated EGFR protein induced by LPA was blocked under BB94 treatment (Figures 2F, G). This result indicates that MMPs are critical for EGFR transactivation in response to LPA.

### LPA enhances geminin expression *via* LPAR_1/3_/MMPs/EGFR/PI3K/mTOR pathway

The biological functions of LPA are driven by transmembrane signaling through specific G protein-coupled receptors (van Corven et al., 1989), and transcriptomic data revealed that LPA receptor (LPAR) genes are expressed *via* a cell type-selective manner (Reinartz et al., 2019). Notably in this regard, the qRT-PCR analysis revealed that LPAR_1_ and LPAR_3_ had the same (higher) expression than the other four LPA receptors in OVCAR5 cells (Figure 2H). The addition of LPAR_1/3_ inhibitor Ki16425 significantly inhibited geminin expression induced by LPA (Figures 2I, J). This result indicates that LPA regulates geminin expression through its receptors LPAR1/3.

Having confirmed that LPA activates the MMPs/EGFR pathway, whether inhibition of the MMPs/EGFR pathway was selective against geminin expression was investigated. The analysis showed that both MMPs inhibitor BB94 and EGFR inhibitor AG1478 prevented LPA-induced geminin expression (Figures 2I, J). These results indicate that the MMPs/EGFR pathway is activated to mediate geminin expression under LPA stimulus. To confirm whether the traditional EGFR signaling pathway was implicated in geminin overexpression, OVCAR5 cells were pretreated with LY294002 and rapamycin to inhibit PI3K and mTOR biological activity, respectively. The analysis of immunoblotting showed that the PI3K inhibitor LY294002 and mTOR inhibitor rapamycin significantly eliminated the upregulation of geminin protein stimulated with LPA in ovarian cancer cells, whereas DMSO had no effect (Figures 2I, J). Taken together, these data suggest that LPA promotes geminin expression *via* the LPAR_1/3_/MMPs/EGFR/PI3K/mTOR pathway in HGSOC cells.

### Aurora-A correlates with geminin in mRNA and protein levels in human ovarian cancer

Enhanced protein synthesis and stabilization are the two main regulatory mechanisms to increase protein expression. Aurora-A kinase was identified to enhance geminin stabilization by phosphorylating geminin on Thr25 in the M phase (Tsunematsu et al., 2013). This means that Aurora-A kinase may be able to regulate geminin stability in HGSOC cells. To address this, the TCGA ovarian cancer data were utilized to explore the correlation between geminin and Aurora-A. A volcano plot analysis of 17,429 genes based on attribute gene *GMNN* was separated into two groups with a significant differential correlation (Supplementary Figures S2A, B). Spearman’s rank correlation coefficient analysis revealed a significant correlation between the expression of *GMNN* and *AURKA* (Supplementary Figure S2C). In addition, the PPI network revealed that the Aurora-A protein had known experimental and text-mining interactions with the geminin protein (PPI enrichment *p* < 0.05) (Supplementary Figure S2D, Supplementary Table S4). Interestingly, homologs were co-expressed in other organisms (data not shown). Collectively, these results imply that Aurora-A correlates with geminin in mRNA and protein levels in human ovarian cancer.

### LPA potentiates geminin protein stability *via* EGFR/Aurora-A^Thr288^ axis in HGSOC cells

**FIGURE 3:**
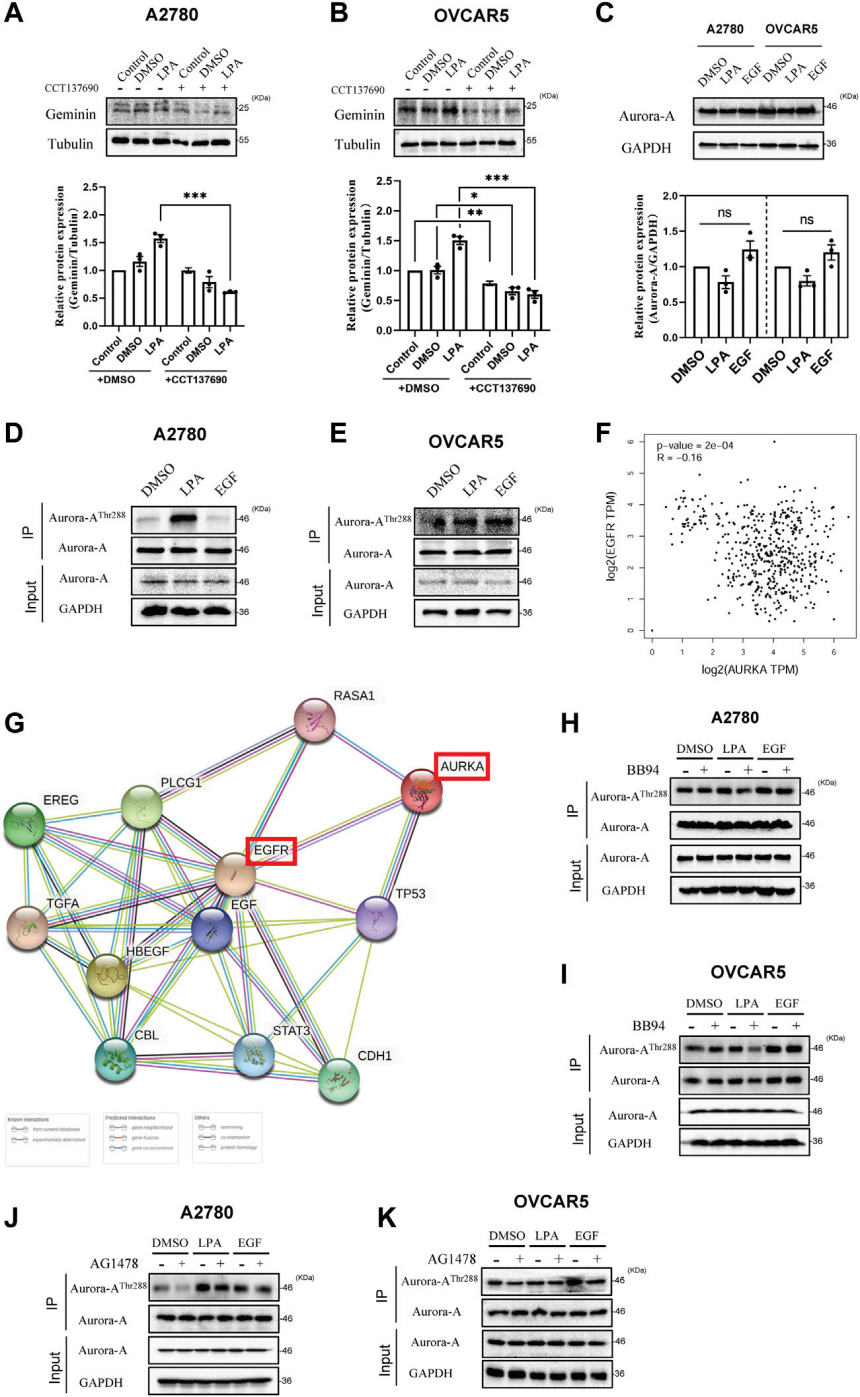
LPA potentiates geminin stability by targeting Aurora-A^Thr288^ in HGSOC cells. **(A,B)** Immunoblotting analysis of geminin protein levels in A2780 cells **(A)** and OVCAR5 cells **(B)** after pretreated with or without CCT137690 (25 μM) for 120 min, and then stimulated with 0.1% DMSO (vehicle) or 10 μM LPA. Mean ± SEM, *n* = 3, VNOVA, Dunnett’s Multiple Comparison Test, *p < 0.05, **p < 0.01, ***p < 0.001. **(C)** IB analysis of Aurora-A protein levels in A2780 cells and OVCAR5 cells. Mean ± SEM, *n* = 3, VNOVA, Dunnett’s Multiple Comparison Test, ns, non-significant. **(D,E)** Immunoprecipitation analysis with anti-phospho-Aurora Kinase (Thr288) antibodies in A2780 cells **(D)** and OVCAR5 cells **(E)** treated with 0.1% DMSO (vehicle), 10 μM LPA or 10 ng/mL EGF for 5 min (A2780 cells) or 30 min (OVCAR5 cells). **(F)** Correlation of gene AURKA and EGFR in TCGA ovarian cancer cohort. log2 fold changes of gene expression on the axis, and the Spearman Method was used for calculating the correlation coefficient. **(G)** The protein-protein associations between Aurora-A and EGFR protein. Data from STRING. **(H–K)** IP analysis with anti-phospho-Aurora Kinase (Thr288) antibodies in A2780 cells **(H,J)** and OVCAR5 cells **(I,K)** after pretreated with or without 10 μM BB94 **(H,I)** or 10 μM AG1478 **(J,K)** for 30 min, then treated with 0.1% DMSO (vehicle), 10 μM LPA or 10 ng/mL EGF for 5 min (A2780 cells) or 30 min (OVCAR5 cells).

Geminin expression was blocked by treating HGSOC cells (A2780 and OVCAR5) with CCT137690 to inhibit Aurora-A kinase activity (Figures 3A, B, compare lanes 1 and 4). Together with the previous report (Tsunematsu et al., 2013), these results indicate that Aurora-A enhances geminin protein stability in HGSOC cells. In addition, LPA-recruited geminin expression was notably abolished by CCT137690, indicating that LPA potentiates geminin stability by targeting Aurora-A in HGSOC cells (Figures 3A, B, compare lanes 3 and 6). However, there were no obvious differences in Aurora-A expression with or without LPA or EGF stimulus (Figure 3C). Indeed, the kinase Aurora-A exerts its biological activity through a phosphorylation state. The analysis of immunoprecipitation showed that Aurora-A^Thr288^ protein levels were markedly increased with LPA stimulus, suggesting LPA signal activates Aurora-A phosphorylation on Thr288 in HGSOC cells (Figures 3D, E). There were also significant differences in the expression of phosphorylated Aurora-A between the EGF groups and the control DMSO (Figure 3E). To further understand the effect of EGFR on Aurora-A phosphorylation, a correlation expression of *AURKA* and *EGFR* was analyzed from the TCGA ovarian cancer data set. Spearman’s rank correlation coefficient analysis manifested a strong correlation between the expression of *AURKA* and *EGFR* (Figure 3F). In addition, the PPI network revealed that Aurora-A protein had known experimental, curated database and text-mining interactions with EGFR protein (PPI enrichment *p* < 0.001) (Figure 3G, Supplementary Table S5). These results indicate that Aurora-A correlates with EGFR in mRNA and protein levels in human ovarian cancer. To validate the direct interaction between Aurora-A and EGFR, HGSOC cells were treated with the inhibitors BB94 and AG1478. The data showed that Aurora-A^Thr288^ protein levels decreased under treatment with the above inhibitors, implying that LPA phosphorylates Aurora-A on Thr288 through the MMPs/EGFR axis (Figures 3H–K). Overall, these data indicate that LPA potentiates geminin stability *via* the EGFR/Aurora-A^Thr288^ axis in HGSOC cells.

### Aurora-A predicts clinical outcomes of ovarian cancer patients

The expression level of Aurora-A was defined to further investigate the role of HGSOC. Notably, *AURKA* transcript levels were higher in most cancer tissues than in normal tissues (Supplementary Figure S3A). Additionally, compared with normal ovarian tissues, ovarian cancer tissues exhibited significantly higher *AURKA* transcript levels (*p* < 0.001) (Supplementary Figure S3B). However-, there were no significant differences among different ovarian pathological stages (*p* = 0.247) (Supplementary Figure S3C). Moreover, the IHC analysis revealed that the Aurora-A protein was barely detectable in normal ovarian tissues, while a stronger signal was detected in cancer tissues (Supplementary Figure S3D). Furthermore, a correlation between Aurora-A expression and disease progression was identified in a sample of 1656 ovarian cancer patients with available clinical data. In the multivariate analysis, the data set was based on overall survival, with an HR of 1.28 and a CI of 95%. The median survival in the high Aurora-A expression cohort was 40.1 months, compared with 48.37 months in the low expression cohort. The Kaplan–Meier plot demonstrated that the survival rate was significantly lower in patients with high Aurora-A expression than in patients with low Aurora-A expression (*p* < 0.001) (Supplementary Figure S3E). These results indicate that Aurora-A overexpression is associated with poor prognosis in human ovarian cancer. In summary, these findings imply that Aurora-A expression levels increase in human ovarian cancer tissue and are positively correlated with disease progression in human ovarian cancer.

### LPA signaling pathway induces DNA synthesis in HGSOC cells

GSEA was performed from the TCGA ovarian cancer database on the LinkedOmics website to evaluate the effects of LPA-activated geminin expression in HGSOC development. GO analysis revealed that the paramount biological process of gene *GMNN* was chromosome-related, such as chromosome segregation, mitotic cell cycle transition, DNA replication, translational elongation, protein location to chromosome, telomere organization, and double-strand break repair (Supplementary Figure S4). GO analysis on molecular function demonstrated that the principal function of gene *GMNN* was also DNA-related, such as structural constituent of the ribosome, single-stranded DNA binding, catalytic activity acting on DNA, damaged DNA binding, and nucleosome binding (Supplementary Figure S5). KEGG pathway analysis manifested that *GMNN* upregulated genes implicated in cell cycle, spliceosome, DNA replication, proteasome, pyrimidine metabolism, ribosome, Fanconi anemia pathway, oxidative phosphorylation, mismatch repair, nucleotide excision repair, base excision repair, and oocyte meiosis (Supplementary Figures S6A, B, Supplementary Table S6). Moreover, the cell cycle enrichment plot indicated that the most enriched gene is proliferating cell nuclear antigen (PCNA), a well-known marker protein as a DNA sliding clamp for DNA polymerase *δ* and as an essential component for eukaryotic chromosomal DNA replication and repair (Supplementary Figure S6C, Supplementary Table S7).

**FIGURE 4:**
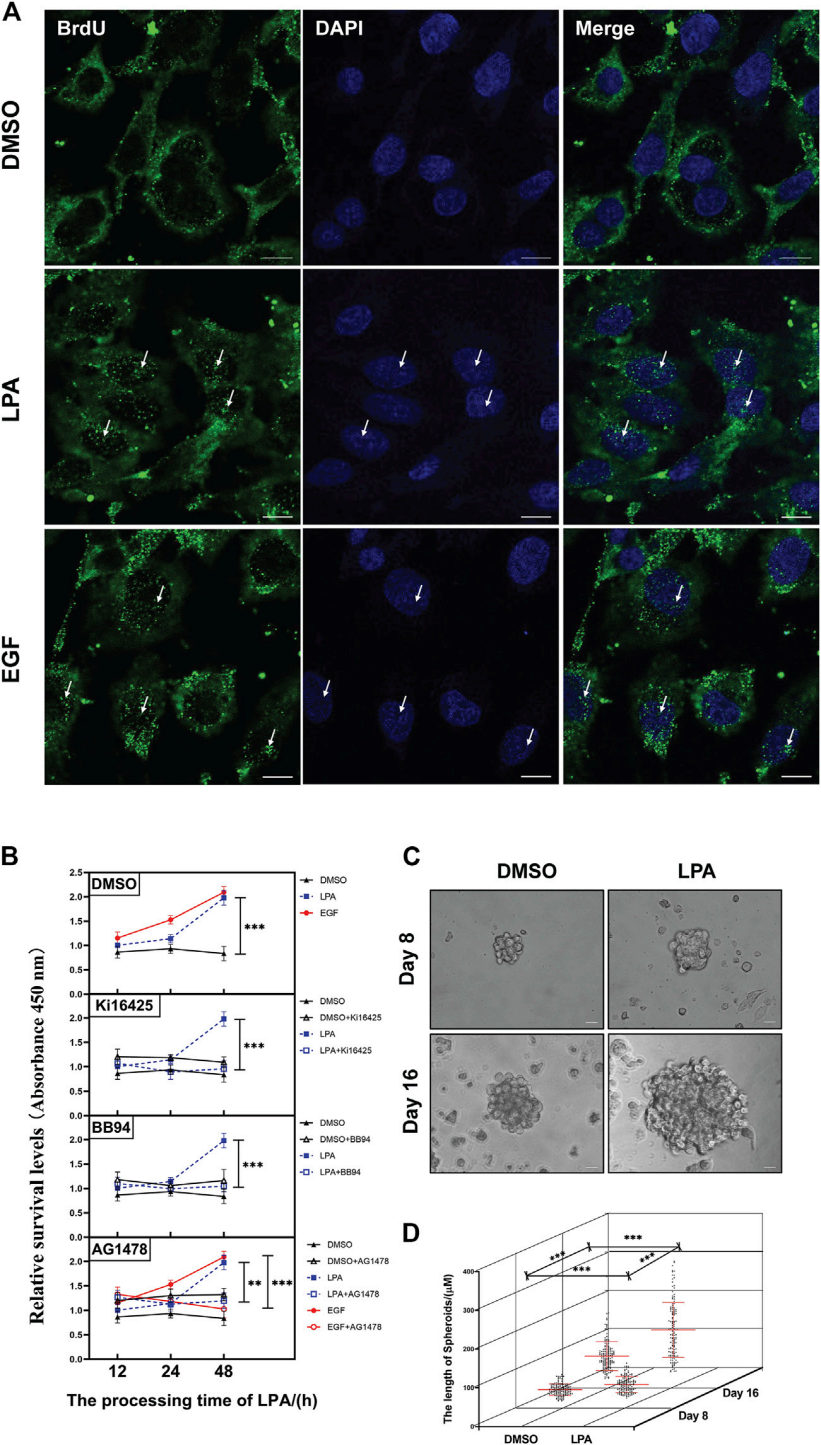
LPA signal mediates DNA replication, cell-cycle progression, and cell proliferation of HGSOC cells. **(A)** Representative images of immunolluorescence staining with anti-BrdU (green) and DAPI/nuclei (blue). Some increased green puncta in the nucleus are identified with white arrows. OVCAR5 cells. Scale bar 10 μm. All images were obtained with the same acquisition conditions. **(B)** The cell proliferation of OVCAR5 cells. Cells were pretreated with specified inhibitors for 30 min and then grown in 0.1% DMSO, 10 µM LPA or 10 ng/ml EGF. Mean ± SEM, *n* = 3, VNOVA, Dunnett’s multiple comparison test, **p < 0.01, ***p < 0.001. **(C)** Representative phase-contrast images of bioreactor expanded OVCARS cells at indicated days of cultivation. Scale bar 20 μm. **(D)** Graphical illustration of the average aggregate size measured at the indicated day of cultivation. Day 8, DMSO *n* = 140, LPA *n* = 139; Day 16, DMSO *n* = 153, LPA *n* = 138. Images were analyzed using Nikon NIS Elements D software, mean ± SD, Student’s unpaired *t*-test, ***p < 0.001.

A PPI network was utilized to generate and precisely exhibit the biological effects of geminin. The network demonstrated that the top 50 proteins that had the strongest interaction with geminin could be mainly divided into two groups, DNA replication-related factors and proteasomes (PPI enrichment *p* < 1.0 × 10^−16^) (Supplementary Figure S7, Supplementary Table S8). Indeed, proteasomes are involved in numerous cellular processes, including cell cycle progression, apoptosis, and DNA damage repair. Taken together, these results infer that the protein geminin correlates most strongly with the DNA replication process in human ovarian cancer. Crucially, the immunofluorescence analysis validated this conclusion. The numbers of green puncta in the nucleus were phenomenally increased after treatment with LPA, suggesting that the LPA signaling pathway induces efficient DNA synthesis in HGSOC cells (Figure 4A).

### LPA signaling pathway promotes cell cycle progression and cell proliferation of HGSOC cells

LPA is a serum phospholipid with growth-factor-like activities in ovarian cancer (Hu et al., 2001). The PPI network of geminin showed there are 41 proteins implicated in cell cycle progression of the top 50 proteins (Supplementary Figure S7, Supplementary Table S8). In addition, the FCM assay showed that LPA stimulus increased the DNA content of OVCAR5 cells in a time-dependent manner, and inhibition of the LPAR_1/3_/MMPs/EGFR/PI3K/mTOR pathway notably abolished LPA-induced DNA increases (Supplementary Figure S8). Moreover, the CCK-8 assay showed that LPA stimulus increased the cell proliferation of OVCAR5 cells, and the inhibitors, such as Ki16425, BB94, and AG1478, significantly blocked the cell proliferation induced by LPA (Figure 4B). The further 3D assay showed a larger than 1.2/1.8-fold increase in the size of tumor spheroids under LPA stimulus (Figures 4C, D). Based on the above findings, the LPA signaling pathway activates cell cycle progression and cell proliferation of HGSOC cells.

## Discussion

Apoptosis is an evolutionary homeostatic process that is responsible for programmed cell death during normal eukaryotic development. It is controlled by the Bcl 2 family proteins (anti-apoptotic proteins and pro-apoptotic proteins) and the downstream caspases signaling pathway. Targeting autophagy activity to promote apoptosis is shown as a potential therapeutic strategy for cancer treatment (Tang et al., 2022). Analysis of haploinsufficiency has suggested that the autophagic pathway is significantly disrupted through gene deletion, such as the *BENC1* and *LC3* genes that were mono-allelically deleted in HGSOC patients (Delaney et al., 2017). The former studies described that geminin depletion results in DNA-damaging apoptosis in malignant cancer cells (Zhu and Depamphilis, 2009). In geminin-depleted HGSOC cells, we find that the expression levels of proteins LC3B and Bcl 2 are decreased while the expression levels of Bax, caspase 9, caspase 3, and PARP are increased and accompanied by increased ROS production and apoptosis activity (Figure 1). The further 3D experiments clearly show the apoptosis effect of geminin depletion in HGSOC cells (Figures 1D, E). These data disclose that geminin depletion-induced ROS production may be the key factor for the apoptosis of HGSOC cells. Our work supplements a basic understanding of the mechanism of apoptosis and suggests novel opportunities to develop biomedical therapy to regulate HGSOC cell apoptosis.

LPA is a simple bioactive molecule that can modulate cell progression (Gschwind et al., 2002) and metastasis (Gschwind et al., 2003; Guo et al., 2015) *via* multiple signaling pathways. Dysregulation in LPA signal transduction leads to oncogenic transformation (Mills and Moolenaar, 2003; Ren et al., 2019). LPA is reported to be increased in ovarian cancer patients (Xu et al., 1995; Xu et al., 1998; Baker et al., 2002; Mills and Moolenaar, 2003; Jesionowska et al., 2015). Recent studies show that LPA regulates cytosolic NHERF1 to chemotactic cell migration of ovarian cancer cells (Oh et al., 2017). Nonetheless, the intracellular mechanism underlying the LPA-induced progression of ovarian cancer has remained unclear. Our findings reveal that LPA stimulus increases geminin mRNA transcription and protein translation in HGSOC cells in a time-dependent manner (Figures 2A–C). EGFR may be transactivated by GPCRs for regulating DNA expression and the cell cycle (Daub et al., 1996; Kalmes et al., 2000; Shah et al., 2003; Alsahafi et al., 2020), as well as by LPA and S1P for mediating cancer pathophysiology (Gschwind et al., 2002; Deng et al., 2004; Sukocheva et al., 2006; Tveteraas et al., 2016). It was reported that the PI3K/mTOR signaling pathway could regulate its targets, such as eukaryotic initiation factor 4E (eIF4E)-binding protein (4E-BP) and ribosomal protein S6 kinase (S6K), which are related to cell cycle, cell survival, and metastasis (Granville et al., 2006; Jiang and Liu, 2008). Dysregulation of PI3K/mTOR may result in cancer tumorigenesis. Pharmacological inhibition experiments was indicated that LPA enhances geminin expression by activating the LPAR_1/3_/MMPs/EGFR/PI3K/mTOR pathway in HGSOC cells (Figures 2D–J).

Aurora kinases are found in *Drosophila*, where they are shown to mediate centrosome separation (Glover et al., 1995). Aurora-A is the first member identified to be related to cancer progression (Damodaran et al., 2017) and recognized as a potential therapeutic target (Cheng et al., 2019; Adhikari et al., 2020; Chen et al., 2021; Sun et al., 2021). Here, relying on the analysis of public databases including the TCGA and HPA, we found that upregulation of mRNA level and protein level of Aurora-A occurred in human ovarian cancer tissues, which correlates with disease progression (Supplementary Figure S3) and is consistent with previous studies (Yang et al., 2004; Chung et al., 2005; Jr et al., 2007). However, how Aurora-A is activated is not fully understood. Some activators of Aurora-A are known, including the LIM-domain protein Ajuba (Goyal et al., 1999; Hirota et al., 2003), the microtubule-associated protein TPX2 (Eyers et al., 2003), p21-activated protein kinase 1 (Pak1) (Zhao et al., 2005), thyroid hormone receptor-associated protein complex component/methyl-CpG binding endonuclease TRAP220/MED1 (Udayakumar et al., 2006), EGFR (Hung et al., 2008; Lai et al., 2010), ubiquitin-specific processing protease-7 (USP7) (Giovinazzi et al., 2013), a classic tumor suppressor p53 (Yang et al., 2018), heterogeneous nuclear ribonucleoprotein Q1 protein (HnRNP Q1) (Lai et al., 2018), the receptor of activated C-kinase1 (RACK1) (Shen et al., 2019), the NE proteins containing the LEM domain LEM4 (Gao et al., 2018), and KIAA1529 (Qiao et al., 2022). Most notably, EGF can increase *Aurora-A* gene expression through the nuclear EGFR/STAT5 signaling pathway and ultimately lead to chromosome instability and tumorigenesis (Hung et al., 2008). The increased expression of Aurora-A in cancers occurs through gene amplification and RNA transcriptional upregulation (Marumoto et al., 2005).

In this work, we show that LPA phosphorylates Aurora-A on Thr288 *via* EGFR transactivation and does not alter Aurora-A expression (Figure 3). Aurora-A kinase is proposed to play a role in protein stabilization (Marumoto et al., 2005). Crucially, Aurora-A is shown to phosphorylate geminin on Thr25 to protect it from APC/C-dependent proteolysis during the M phase (Tsunematsu et al., 2013). Relying on the GESA analysis, we show that expression of geminin correlates with Aurora-A in mRNA and protein levels in human ovarian cancer (Supplementary Figure S2), and the LPA signal stabilizes the geminin protein through the EGFR/Aurora-A^Thr288^ axis (Figure 3).

**FIGURE 5:**
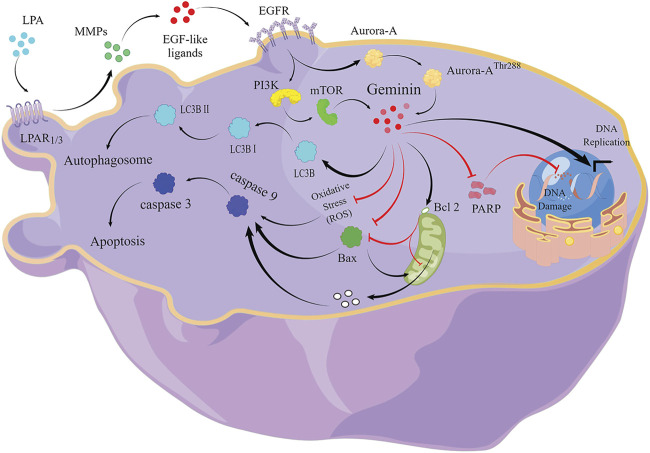
The signaling pathway of LPA-mediated DNA replication initiation, cell-cycle progression, and autophagy in HGSOC cells.

In summary, we confirm that the LPA signal activates geminin expression *via* the LPAR_1/3_/MMPs/EGFR/PI3K/mTOR pathway and enhances geminin stability *via* the EGFR/Aurora-A^Thr288^ pathway (Figure 5). Increased geminin selectively enhances autophagy activity, suppresses ROS production and apoptosis of HGSOC cells, and maintains DNA high-speed replication in the subsequent proliferation (Figure 4). Our results indicated that disrupting the critical signaling pathways may serve as a novel therapeutic strategy for HGSOC treatment.

## Data Availability

The original contributions presented in the study are included in the article/Supplementary Material; further inquiries can be directed to the corresponding author.
