# Supplementary material for: Lysophosphatidic acid suppresses apoptosis of high-grade serous ovarian cancer cells by inducing autophagy activity and promotes cell-cycle progression via EGFR-PI3K/Aurora-AThr288-geminin dual signaling pathways
Source: Front Pharmacol. 2022 Dec 19;13:1046269. doi: 10.3389/fphar.2022.1046269 (PMC9806123; doi:10.3389/fphar.2022.1046269)
Supplement: Supplementary file 2 [file DataSheet1.pdf]

Supplemental Figure legends

Figure S1 Geminin expression levels increase in human ovarian cancer tissues compared with normal ovary tissues. (A) The Gene Expression Profile *GMNN* in human tumor tissues and normal tissues. Data are analysed with GEPIA 2 website, matched TCGA normal and GTEx data, using log2(TPM + 1) for log-scale. VNOVA methods. (B) The RNA expression of geminin in human ovarian cancer tissues and normal ovary tissues based on TCGA database search. ***p<0.001, Student's unpaired t-test were used for data analysis. (C) The RNA expression of geminin in different human ovarian cancer stages. (D) The RNA expression of geminin in different human ovarian cancer patient age distribution. The data was extracted from TCGA database. (E) IHC staining showing geminin expression. The data was extracted from HPA. Scale bar, 200 μM.

Figure S2 Aurora-A correlates with Geminin in RNA and protein levels. (A) Volcano-plot analysis for 20032 genes by RNAseq data based on attribute GMNN in TCGA ovarian cancer cohort, log2 fold changes of gene expression on the x-axis, FDR statistical significance (−log10 *p* value) on the y-axis; upregulated (red), downregulated (green) genes. Genes with at least a >1.5 fold change and FDR < 0.01 are displayed. (B) Het map of part positively correlated significant genes and negatively correlated significant genes in TCGA ovarian cancer cohort. Gene expression (n=20,032) is normalized log2 counts/million. (C) Correlation of gene *GMNN* and *AURKA* in TCGA ovarian cancer cohort. log2 fold changes of gene expression on the axis, and the Spearman Method was used for calculating the correlation coefficient. (D) The protein-protein associations between Aurora-A and geminin protein. Data from STRING.

Figure S3 Aurora-A is overexpressed in ovarian cancer tissues and positively correlates with disease progression. (A) The Gene Expression Profile Aurora-A in human tumor tissues and normal tissues. Data are analysed with GEPIA 2 website, matched TCGA normal and GTEx data, using log2(TPM + 1) for log-scale. VNOVA methods. (B) The RNA expression of Aurora-A in human ovarian cancer tissues and normal ovary tissues based on TCGA database. ***p<0.001, Student's unpaired t-test were used for data analysis. (C) The RNA expression of Aurora-A in different human ovarian cancer stages. Data are analysed with GEPIA 2 website, using log2(TPM + 1) for log-scale. (D) IHC staining showing Aurora-A expression. The data was extracted from HPA. Scale bar, 200 μM. (E) Kaplan-Meier curves are made to show the survival rate of 1676 ovarian cancer patiens with high and low *Aurora-A* mRNA expression levels.

Figure S4 The GO Enrichment analysis on Biological Process category of gene *GMNN*. The results were extracted from LinkedOmics publicly portal, carried out with GSEA method and based upon the 17429 unique entrezgene IDs. The GO Slim summary (A) are based upon the 17429 unique entrezgene IDs and the height of the bar represents the number of IDs in the user list and also in the category. 25 positive related categories and 25 negative related categories are identified as enriched categories, in which 40 most significant categories and represetatives in the reduced sets are shown in the Enrichment Results. The normalized enrichment score (NES) is used to evaluate the statistic for examining gene set enrichment results. The Enrichment Result bar chat (B) and DAG plot (C) are based upon the 13310 unique entrezgene IDs.

Figure S5 The GO Enrichment analysis on Molecular Function category of gene *GMNN*. The results were extracted from LinkedOmics publicly portal, carried out with GSEA method and based upon the 17429 unique entrezgene IDs. (A) The GO Slim summaryare on Molecular Function category of *GMNN* based upon the 17429 unique entrezgene IDs and the height of the bar represents the number of IDs in the user list and also in the category. 25 positive related categories and 25 negative related categories are identified as enriched categories, in which 40 most significant categories and represetatives in the reduced sets are shown in the Enrichment Results. The normalized enrichment score (NES) is used to evaluate the statistic for examining gene set enrichment results. The Enrichment Result bar chat (B) and DAG plot (C) are based upon the 11308 unique entrezgene IDs.

Figure S6 The KEGG Enrichment analysis of gene *GMNN*. The results were extracted from LinkedOmics publicly portal, carried out with GSEA method and based upon the 17429 unique entrezgene IDs. The normalized enrichment score (NES) is used to evaluate the statistic for examining gene set enrichment results. The Enrichment Result bar chat (A) and Volcano plot (B) are based upon the 11308 unique entrezgene IDs. (C) KEGG pathway analysis for cell cycle gene sets with *GMNN* based upon the annotated 6751 IDs.

Figure S7 The protein-protein associations of protein geminin. The top 50 proteins that had the strongest interaction with geminin were shown with the highest confidence 0.900. The edges indicate both functional and physical protein associations. Data from STRING.

Figure S8 LPA signal mediates cell-cycle progression of HGSOC cells. (A) Cell cycle diagrams of the samples pretreated with 0.1% DMSO, 10 μM Ki16425, 10 μM BB94, 10 μM AG1478, 10 μM LY294002 or 100 nM Rapamycin for 30 minutes, and stimulated with 10 μM LPA up to 12 hours. (B) Quantificantion of DNA content in A. Data are analyzed with Origin 2021pro, One way VNOVA, Dunnett's Multiple Comparison Test, mean ± SEM, n=3, *p<0.05, **p<0.01, ***p<0.001.

Fig S1

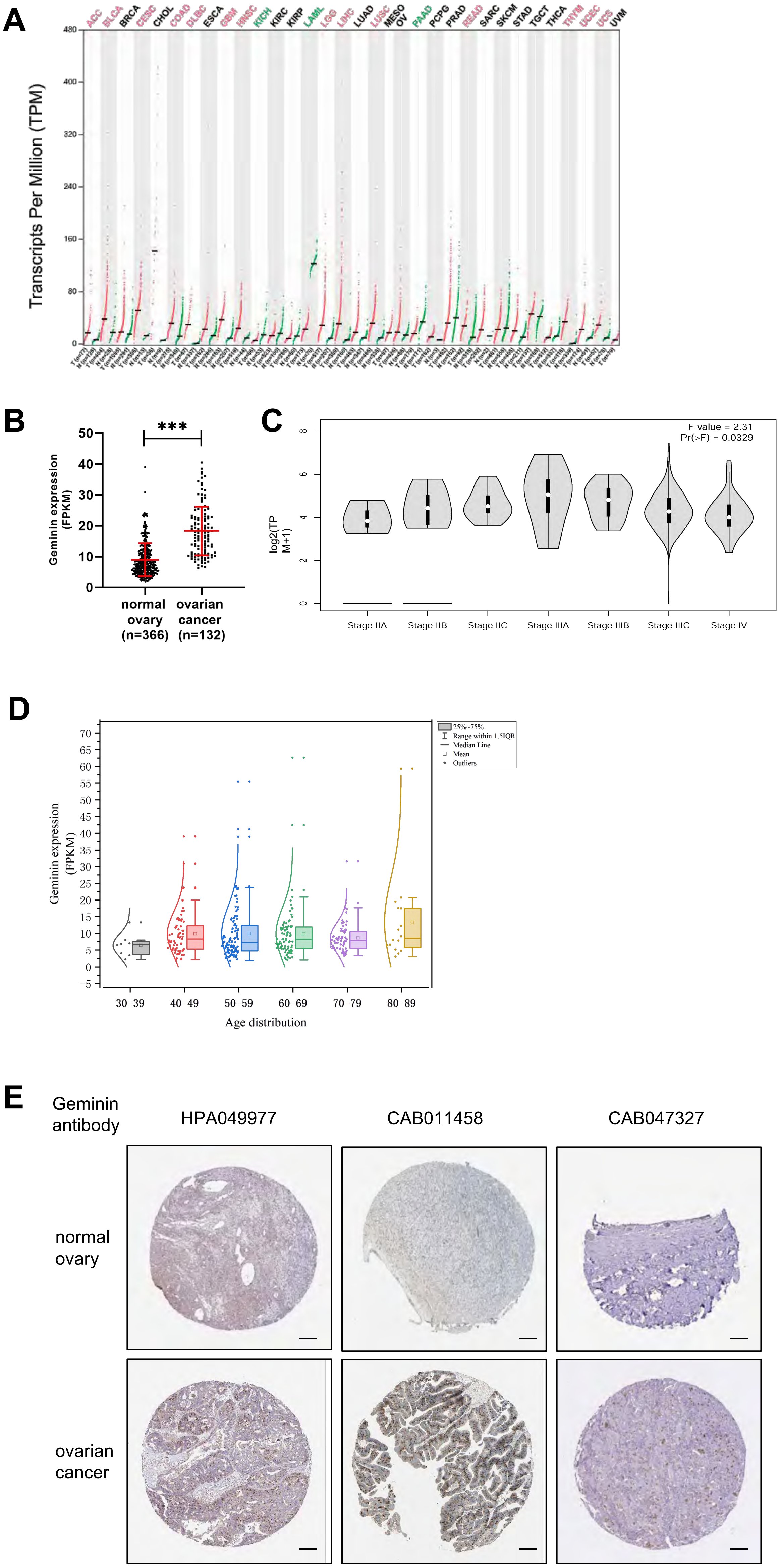
A
Transcripts Per Million (TPM)
ACC BLCA BRCA CESC CHOL COAD DLBC ESCA GBM HNSC KICH KIRC KIRP LAML LGG LIHC LUAD LUSC MESO OV PAAD PCPG PRAD READ SARC SKCM STAD TGCT THCA THYM UCEC UCS UVM
B
Geminin expression (FPKM)
***
normal ovary (n=366)
ovarian cancer (n=132)
C
F value = 2.31
Pr(>F) = 0.0329
log2(TPM+1)
Stage IIA
Stage IIB
Stage IIC
Stage IIIA
Stage IIIB
Stage IIIC
Stage IV
D
25%~75%
Range within 1.5IQR
Median Line
Mean
Outliers
Geminin expression (FPKM)
30~39
40~49
50~59
60~69
70~79
80~89
Age distribution
E
Geminin antibody
HPA049977
CAB011458
CAB047327
normal ovary
ovarian cancer

Fig S2

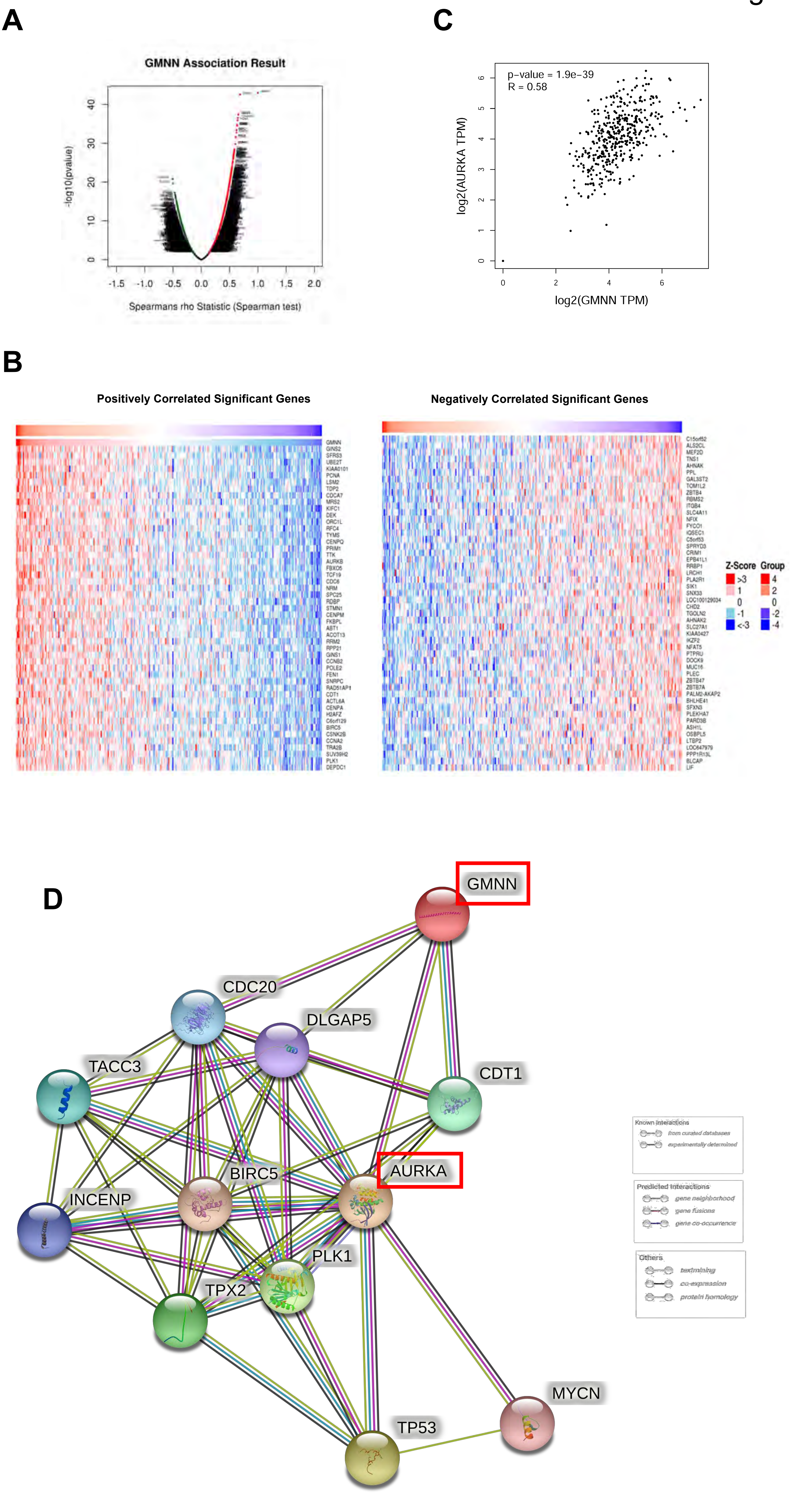
A
GMNN Association Result
-log10(pvalue)
Spearmans rho Statistic (Spearman test)
C
p-value = 1.9e-39
R = 0.58
log2(AURKA TPM)
log2(GMNN TPM)
B
Positively Correlated Significant Genes
GMNN GINS2 SFRS3 UBE2T KIAA0101 PCNA LSM2 TDP2 CDCA7 MRS2 KIFC1 DEK ORC1L RFC4 TYMS CENPQ PRIM1 TTK AURKB FBXO5 TCF19 CDC6 NRM SPC25 RDBP STMN1 CENPM FKBPL ABT1 ACOT13 RRM2 RPP21 GINS1 CCNB2 POLE2 FEN1 SNRPC RAD51AP1 CDT1 ACTL6A CENPA H2AFZ C6orf129 BIRC5 CSNK2B CCNA2 TRA2B SUV39H2 PLK1 DEPDC1
Negatively Correlated Significant Genes
C15orf52 ALS2CL MEF2D TNS1 AHNAK PPL GAL3ST2 TOM1L2 ZBTB4 RBMS2 ITGB4 SLC4A11 NFIX FYCO1 IQSEC1 C5orf53 SPRYD3 CRIM1 EPB41L1 RRBP1 LRCH1 PLA2R1 SIK1 SNX33 LOC100129034 CHD2 TGOLN2 AHNAK2 SLC27A1 KIAA0427 IKZF2 NFAT5 PTPRU DOCK9 MUC16 PLEC ZBTB47 ZBTB7A PALM2-AKAP2 BHLHE41 SFXN3 PLEKHA7 PARD3B ASH1L OSBPL5 LTBP2 LOC647979 PPP1R13L BLCAP LIF
Z-Score
Group
D
GMNN
CDC20
DLGAP5
TACC3
CDT1
BIRC5
AURKA
INCENP
PLK1
TPX2
MYCN
TP53
Known Interactions
from curated databases
experimentally determined
Predicted Interactions
gene neighborhood
gene fusions
gene co-occurrence
Others
textmining
co-expression
protein homology

Fig S3

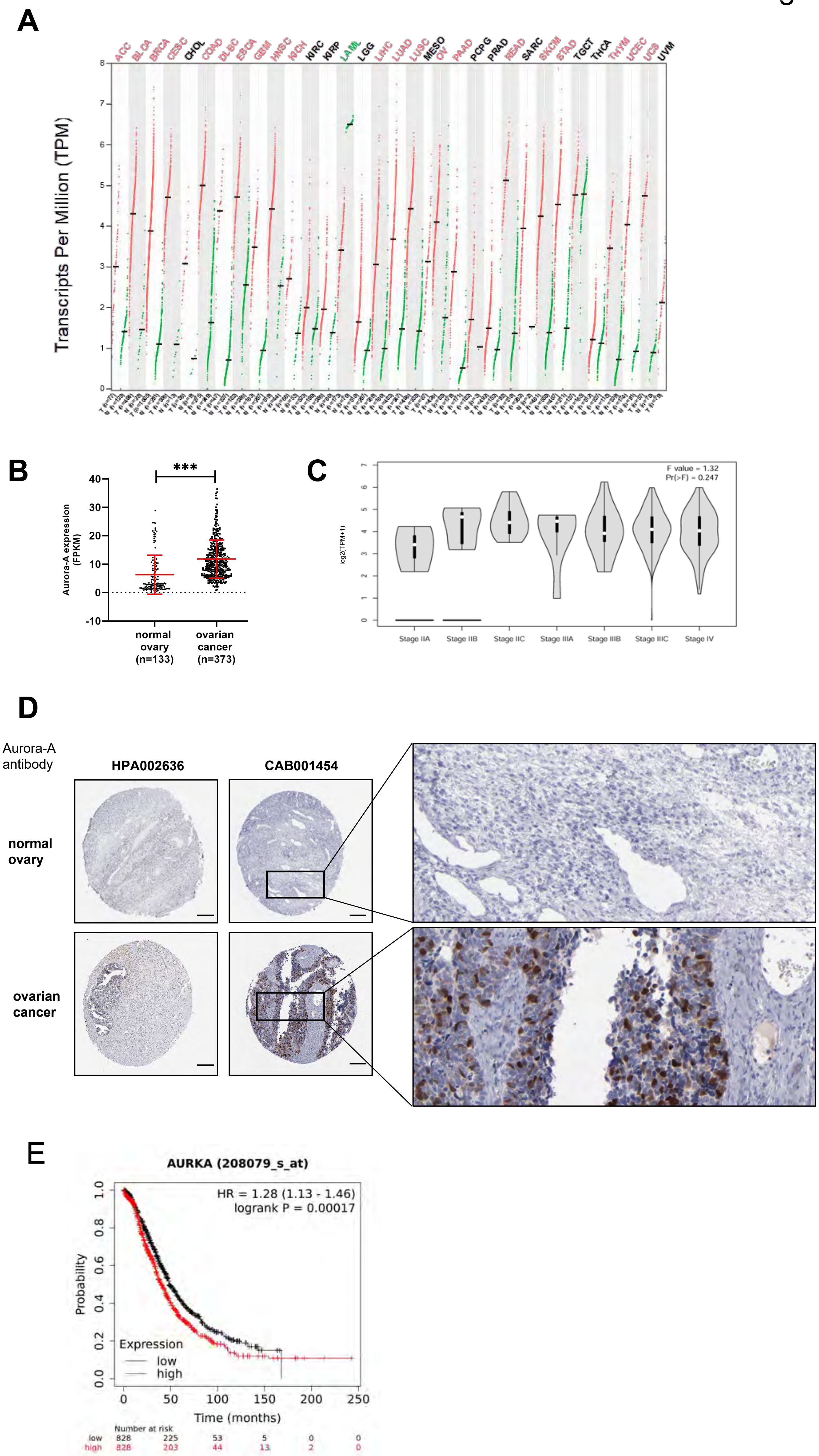
A
ACC BLCA BRCA CESC CHOL COAD DLBC ESCA GBM HNSC KICH KIRC KIRP LAML LGG LIHC LUAD LUSC MESO OV PAAD PCPG PRAD READ SARC SKCM STAD TGCT THCA THYM UCEC UCS UVM
Transcripts Per Million (TPM)
B
***
Aurora-A expression (FPKM)
normal ovary (n=133)
ovarian cancer (n=373)
C
F value = 1.32
Pr(>F) = 0.247
log2(TPM+1)
Stage IIA
Stage IIB
Stage IIC
Stage IIIA
Stage IIIB
Stage IIIC
Stage IV
D
Aurora-A antibody
HPA002636
CAB001454
normal ovary
ovarian cancer
E
AURKA (208079_s_at)
HR = 1.28 (1.13 - 1.46)
logrank P = 0.00017
Probability
Expression
low
high
Time (months)
Number at risk
low 828 225 53 5 0 0
high 828 203 44 13 2 0

A

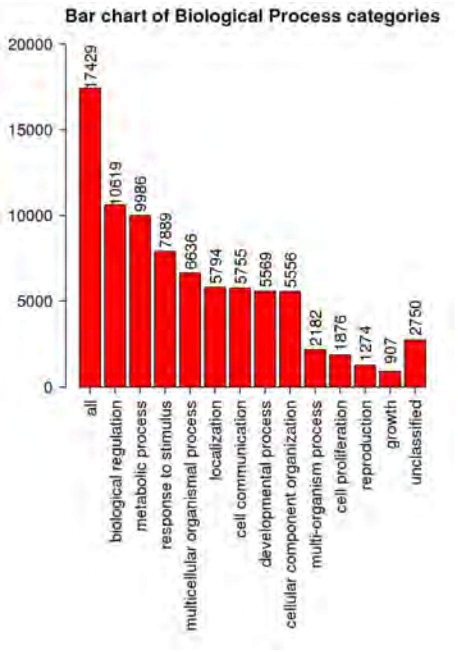
Bar chart of Biological Process categories
20000
15000
10000
5000
0
17429
10619
9986
7889
6636
5794
5755
5569
5556
2182
1876
1274
907
2750
all
biological regulation
metabolic process
response to stimulus
multicellular organismal process
localization
cell communication
developmental process
cellular component organization
multi-organism process
cell proliferation
reproduction
growth
unclassified

B

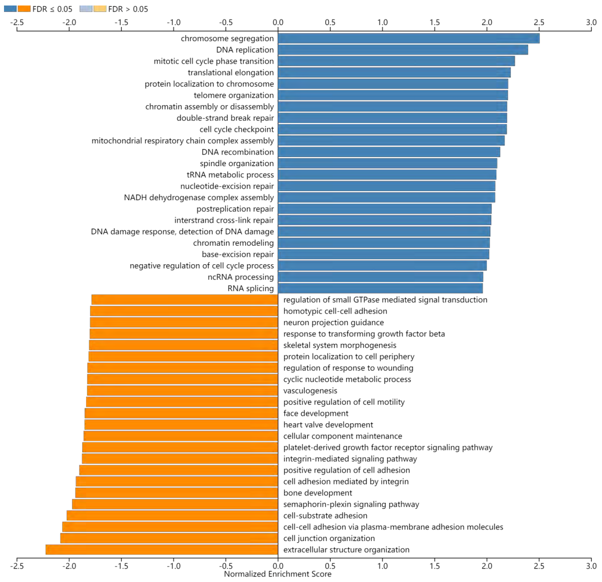
FDR ≤ 0.05
FDR > 0.05
-2.5 -2.0 -1.5 -1.0 -0.5 0.0 0.5 1.0 1.5 2.0 2.5 3.0
chromosome segregation
DNA replication
mitotic cell cycle phase transition
translational elongation
protein localization to chromosome
telomere organization
chromatin assembly or disassembly
double-strand break repair
cell cycle checkpoint
mitochondrial respiratory chain complex assembly
DNA recombination
spindle organization
tRNA metabolic process
nucleotide-excision repair
NADH dehydrogenase complex assembly
postreplication repair
interstrand cross-link repair
DNA damage response, detection of DNA damage
chromatin remodeling
base-excision repair
negative regulation of cell cycle process
ncRNA processing
RNA splicing
regulation of small GTPase mediated signal transduction
homotypic cell-cell adhesion
neuron projection guidance
response to transforming growth factor beta
skeletal system morphogenesis
protein localization to cell periphery
regulation of response to wounding
cyclic nucleotide metabolic process
vasculogenesis
positive regulation of cell motility
face development
heart valve development
cellular component maintenance
platelet-derived growth factor receptor signaling pathway
integrin-mediated signaling pathway
positive regulation of cell adhesion
cell adhesion mediated by integrin
bone development
semaphorin-plexin signaling pathway
cell-substrate adhesion
cell-cell adhesion via plasma-membrane adhesion molecules
cell junction organization
extracellular structure organization
Normalized Enrichment Score

C

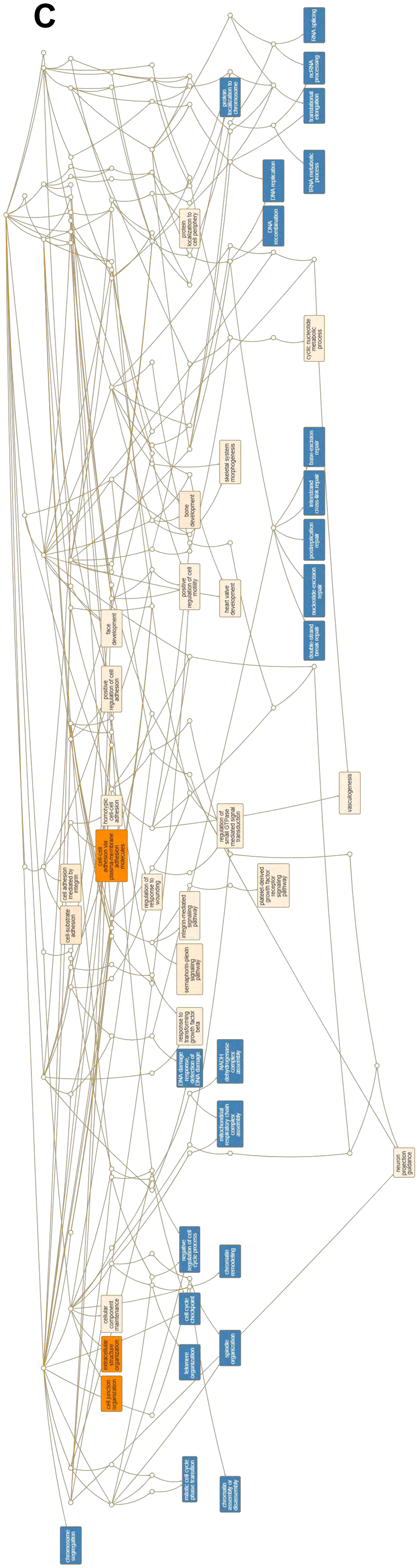

Fig S5

A

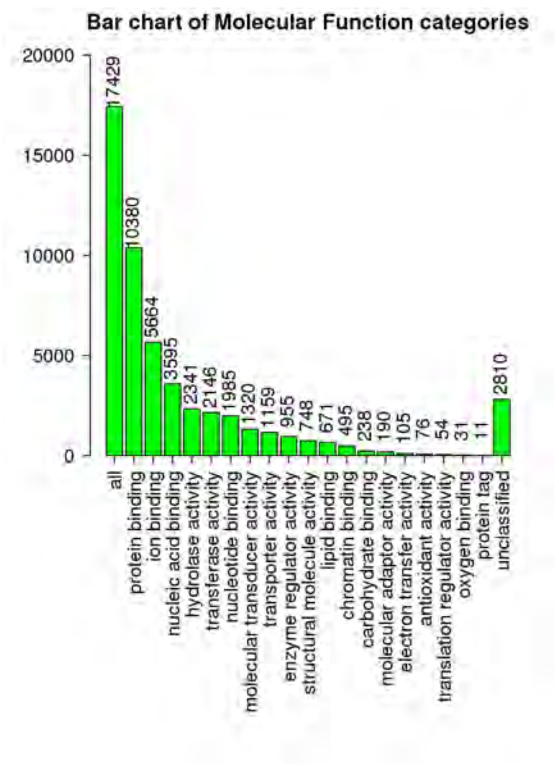

Bar chart of Molecular Function categories
20000
15000
10000
5000
0
17429
10380
5664
3595
2341
2146
1985
1320
1159
955
748
671
495
238
190
105
76
54
31
11
2810
all
protein binding
ion binding
nucleic acid binding
hydrolase activity
transferase activity
nucleotide binding
molecular transducer activity
transporter activity
enzyme regulator activity
structural molecule activity
lipid binding
chromatin binding
carbohydrate binding
molecular adaptor activity
electron transfer activity
antioxidant activity
translation regulator activity
oxygen binding
protein tag
unclassified

B

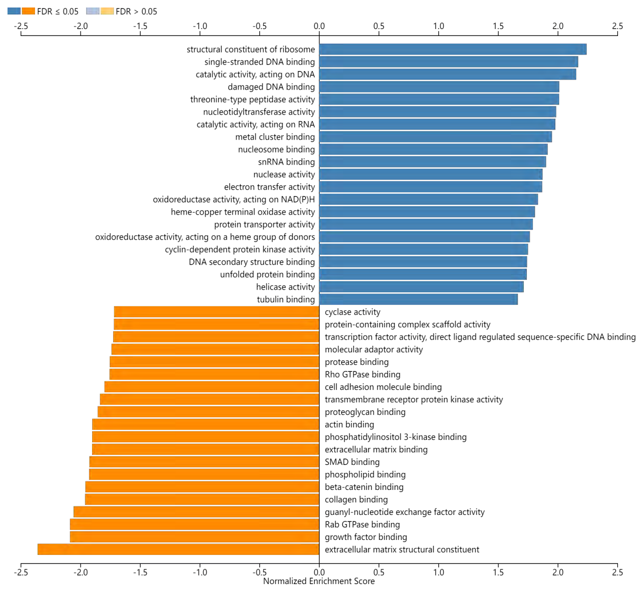

FDR ≤ 0.05
FDR > 0.05
-2.5 -2.0 -1.5 -1.0 -0.5 0.0 0.5 1.0 1.5 2.0 2.5
structural constituent of ribosome
single-stranded DNA binding
catalytic activity, acting on DNA
damaged DNA binding
threonine-type peptidase activity
nucleotidyltransferase activity
catalytic activity, acting on RNA
metal cluster binding
nucleosome binding
snRNA binding
nuclease activity
electron transfer activity
oxidoreductase activity, acting on NAD(P)H
heme-copper terminal oxidase activity
protein transporter activity
oxidoreductase activity, acting on a heme group of donors
cyclin-dependent protein kinase activity
DNA secondary structure binding
unfolded protein binding
helicase activity
tubulin binding
cyclase activity
protein-containing complex scaffold activity
transcription factor activity, direct ligand regulated sequence-specific DNA binding
molecular adaptor activity
protease binding
Rho GTPase binding
cell adhesion molecule binding
transmembrane receptor protein kinase activity
proteoglycan binding
actin binding
phosphatidylinositol 3-kinase binding
extracellular matrix binding
SMAD binding
phospholipid binding
beta-catenin binding
collagen binding
guanyl-nucleotide exchange factor activity
Rab GTPase binding
growth factor binding
extracellular matrix structural constituent
Normalized Enrichment Score

C

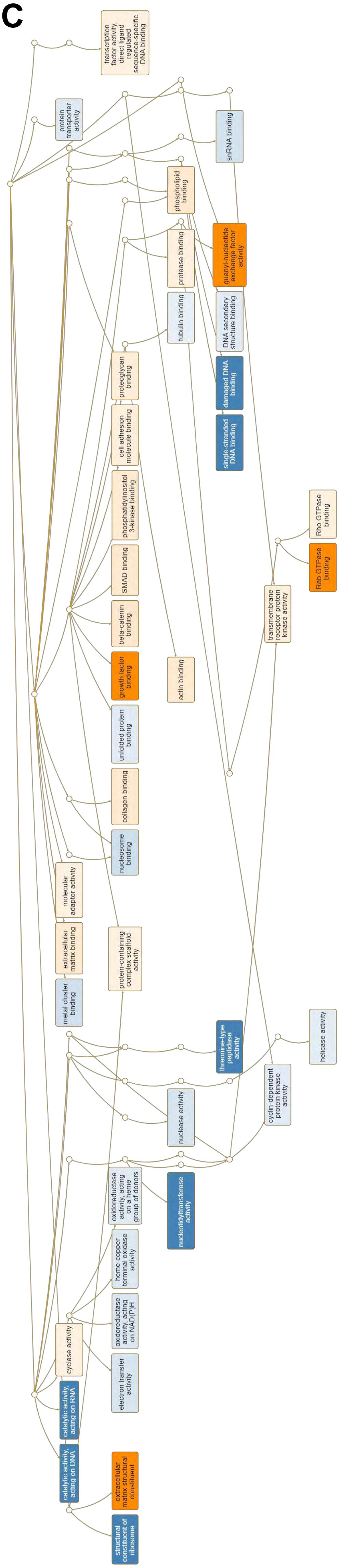

transcription factor activity, direct ligand regulated sequence-specific DNA binding
protein transporter activity
snRNA binding
phospholipid binding
guanyl-nucleotide exchange factor activity
protease binding
tubulin binding
DNA secondary structure binding
damaged DNA binding
single-stranded DNA binding
proteoglycan binding
cell adhesion molecule binding
phosphatidylinositol 3-kinase binding
SMAD binding
beta-catenin binding
growth factor binding
actin binding
unfolded protein binding
collagen binding
nucleosome binding
Rho GTPase binding
Rab GTPase binding
transmembrane receptor protein kinase activity
molecular adaptor activity
extracellular matrix binding
protein-containing complex scaffold activity
metal cluster binding
threonine-type peptidase activity
helicase activity
nuclease activity
cyclin-dependent protein kinase activity
oxidoreductase activity, acting on a heme group of donors
nucleotidyltransferase activity
heme-copper terminal oxidase activity
oxidoreductase activity, acting on NAD(P)H
cyclase activity
electron transfer activity
catalytic activity, acting on RNA
catalytic activity, acting on DNA
extracellular matrix structural constituent
structural constituent of ribosome

Fig S6

A

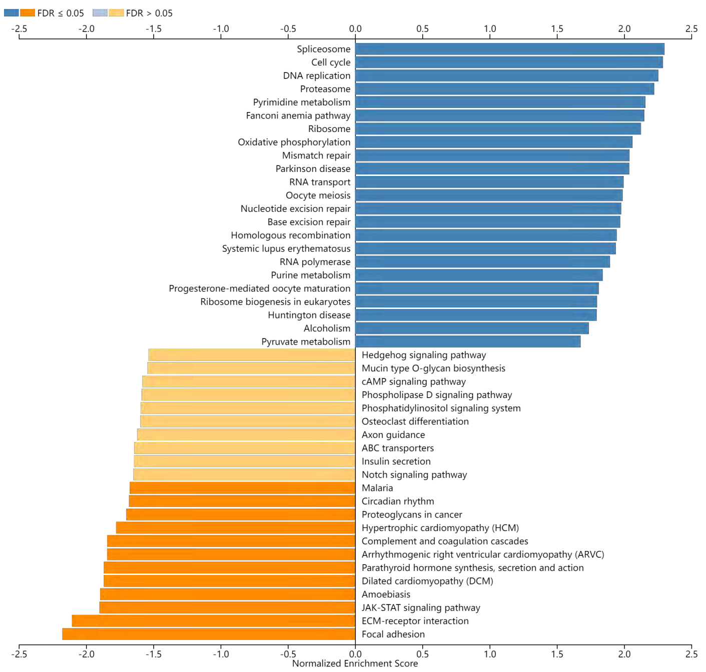
FDR ≤ 0.05
FDR > 0.05
Spliceosome
Cell cycle
DNA replication
Proteasome
Pyrimidine metabolism
Fanconi anemia pathway
Ribosome
Oxidative phosphorylation
Mismatch repair
Parkinson disease
RNA transport
Oocyte meiosis
Nucleotide excision repair
Base excision repair
Homologous recombination
Systemic lupus erythematosus
RNA polymerase
Purine metabolism
Progesterone-mediated oocyte maturation
Ribosome biogenesis in eukaryotes
Huntington disease
Alcoholism
Pyruvate metabolism
Hedgehog signaling pathway
Mucin type O-glycan biosynthesis
cAMP signaling pathway
Phospholipase D signaling pathway
Phosphatidylinositol signaling system
Osteoclast differentiation
Axon guidance
ABC transporters
Insulin secretion
Notch signaling pathway
Malaria
Circadian rhythm
Proteoglycans in cancer
Hypertrophic cardiomyopathy (HCM)
Complement and coagulation cascades
Arrhythmogenic right ventricular cardiomyopathy (ARVC)
Parathyroid hormone synthesis, secretion and action
Dilated cardiomyopathy (DCM)
Amoebiasis
JAK-STAT signaling pathway
ECM-receptor interaction
Focal adhesion
-2.5 -2.0 -1.5 -1.0 -0.5 0.0 0.5 1.0 1.5 2.0 2.5
Normalized Enrichment Score

B

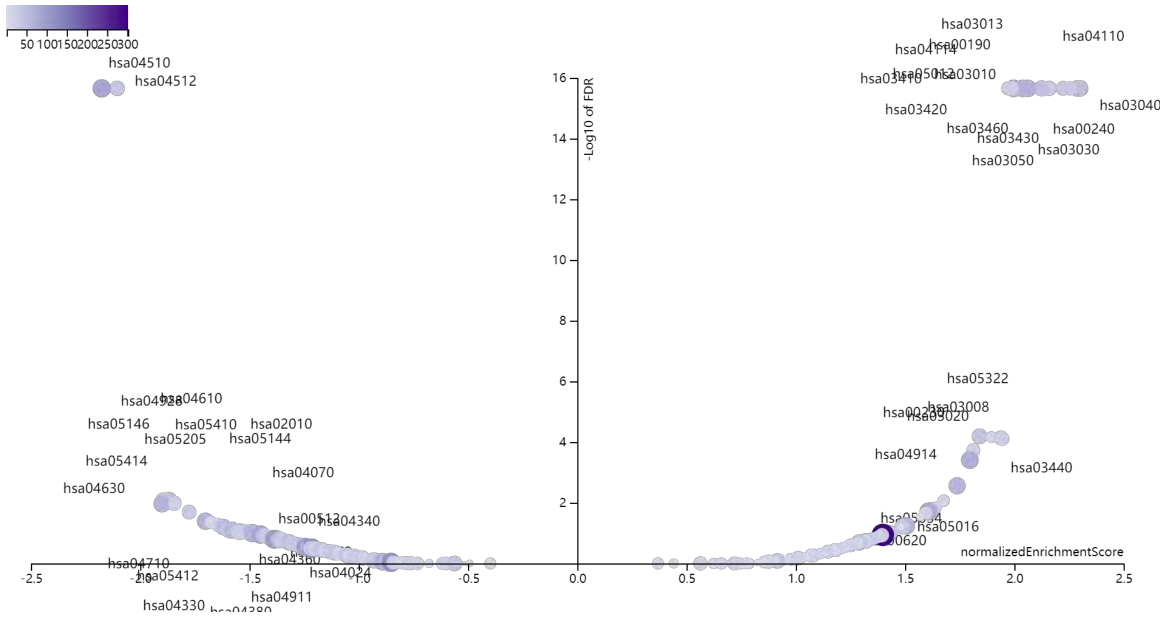
50 100 150 200 250 300
hsa04510
hsa04512
hsa03013
hsa04110
hsa00190
hsa03010
hsa03410
hsa03040
hsa03420
hsa03460
hsa00240
hsa03430
hsa03030
hsa03050
-Log10 of FDR
16
14
12
10
8
6
4
2
hsa05322
hsa03008
hsa03020
hsa04914
hsa03440
hsa05016
normalizedEnrichmentScore
hsa04610
hsa05146
hsa05410
hsa02010
hsa05205
hsa05144
hsa05414
hsa04070
hsa04630
hsa04340
hsa04710
hsa05412
hsa04024
hsa04911
hsa04330
-2.5 -2.0 -1.5 -1.0 -0.5 0.0 0.5 1.0 1.5 2.0 2.5

C

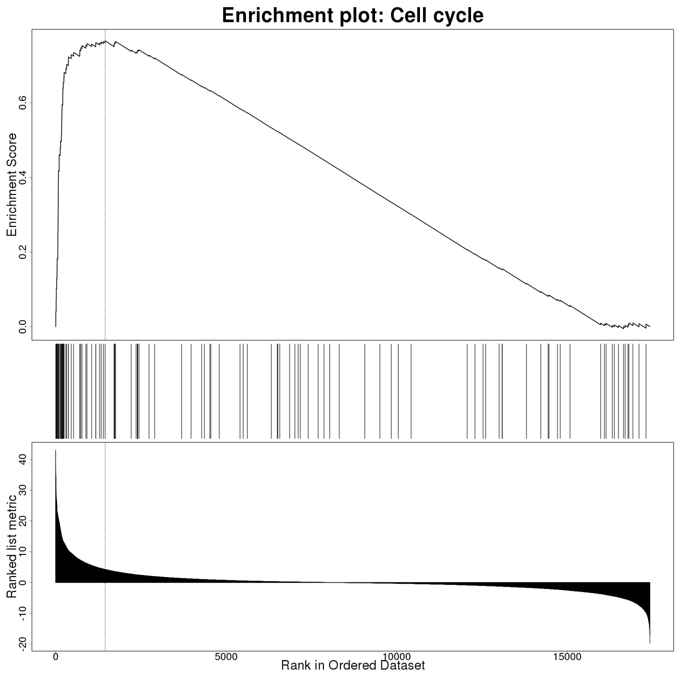
Enrichment plot: Cell cycle
Enrichment Score
0.0
0.2
0.4
0.6
Ranked list metric
-20
-10
0
10
20
30
40
0
5000
10000
15000
Rank in Ordered Dataset

Fig S7

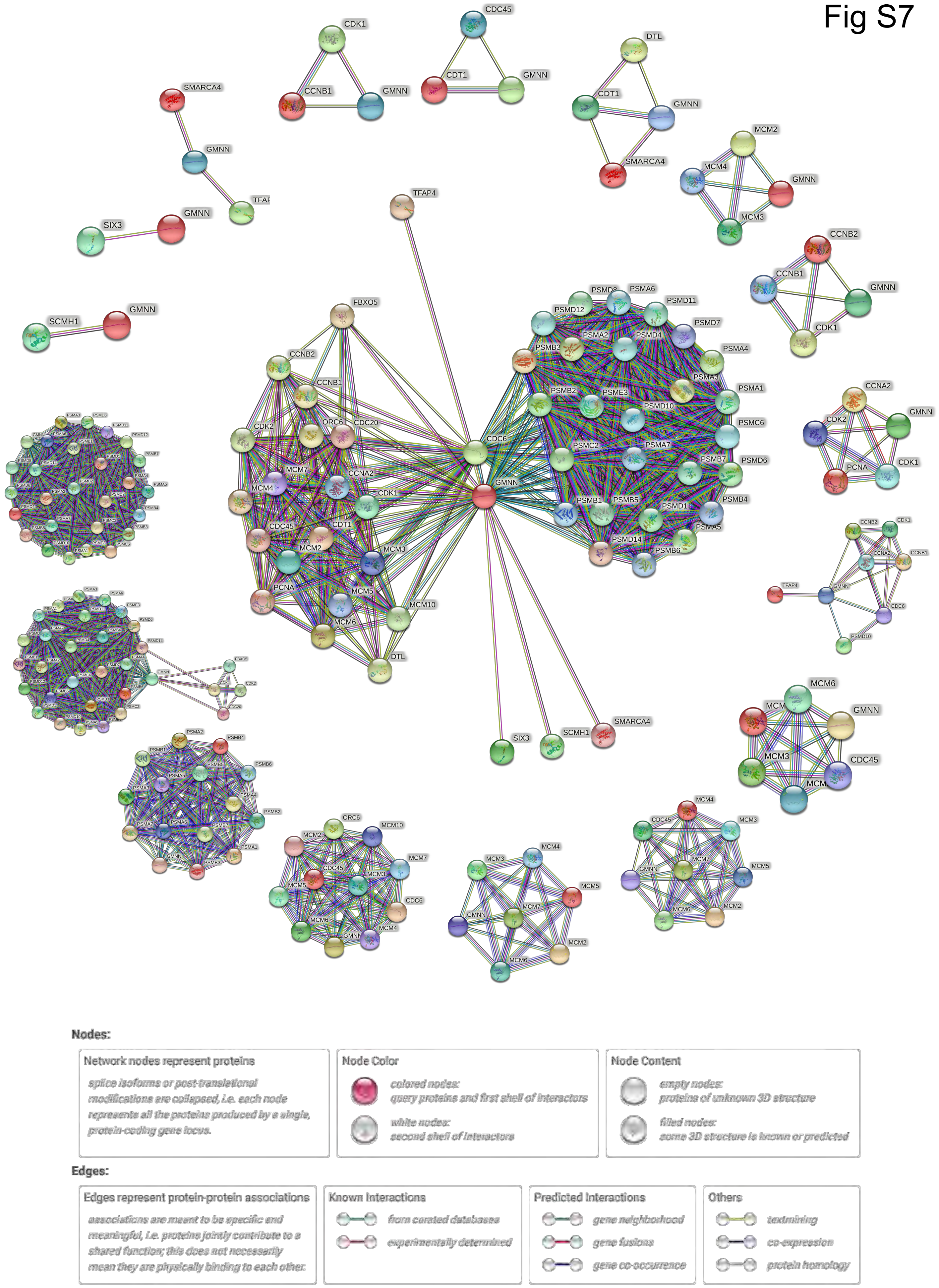

Nodes:
Network nodes represent proteins
splice isoforms or post-translational modifications are collapsed, i.e. each node represents all the proteins produced by a single, protein-coding gene locus.
Node Color
colored nodes:
query proteins and first shell of interactors
white nodes:
second shell of interactors
Node Content
empty nodes:
proteins of unknown 3D structure
filled nodes:
some 3D structure is known or predicted
Edges:
Edges represent protein-protein associations
associations are meant to be specific and meaningful, i.e. proteins jointly contribute to a shared function; this does not necessarily mean they are physically binding to each other.
Known Interactions
from curated databases
experimentally determined
Predicted Interactions
gene neighborhood
gene fusions
gene co-occurrence
Others
textmining
co-expression
protein homology

Fig S8

A

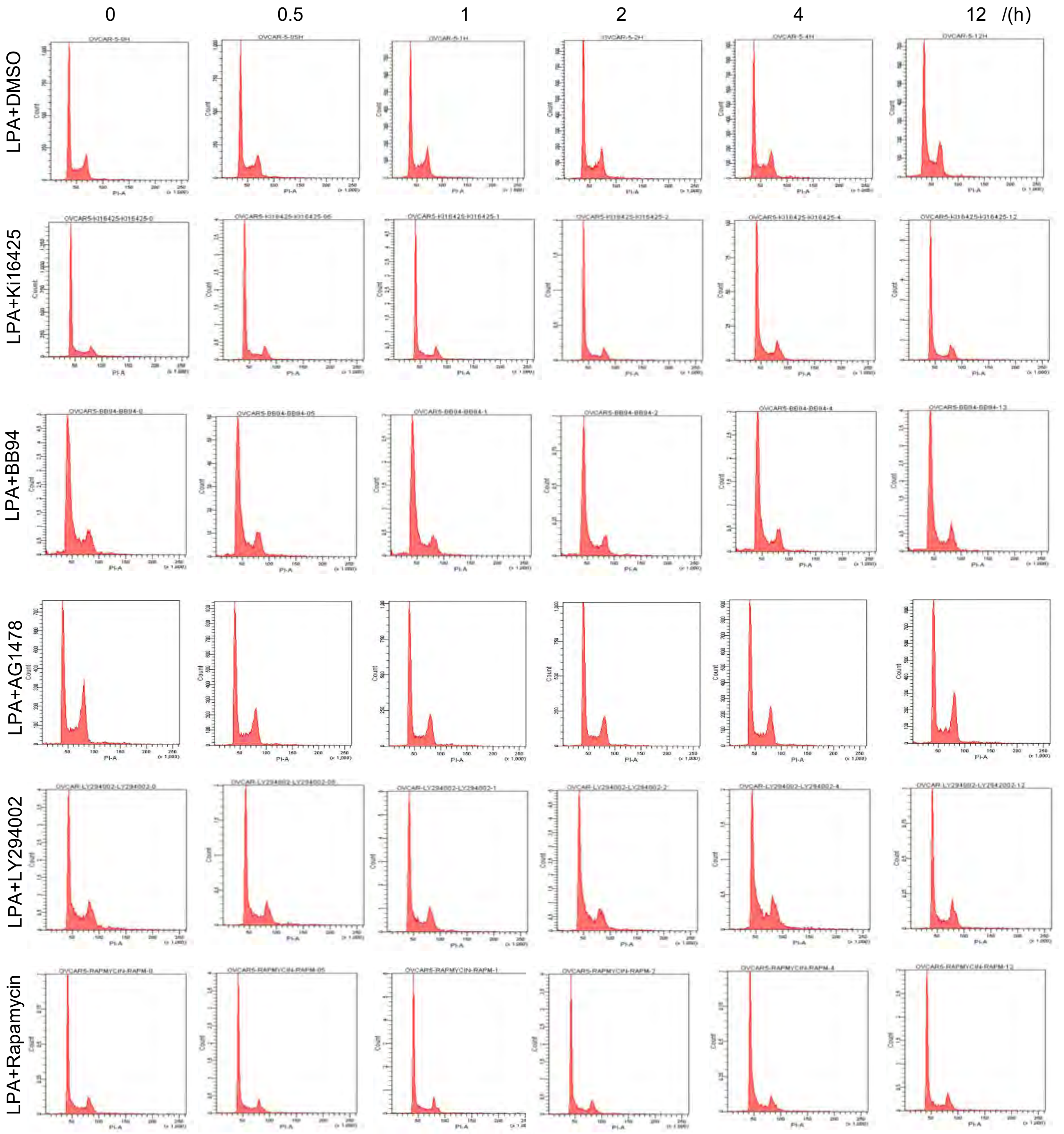
0
0.5
1
2
4
12 /(h)
LPA+DMSO
LPA+Ki16425
LPA+BB94
LPA+AG1478
LPA+LY294002
LPA+Rapamycin

B

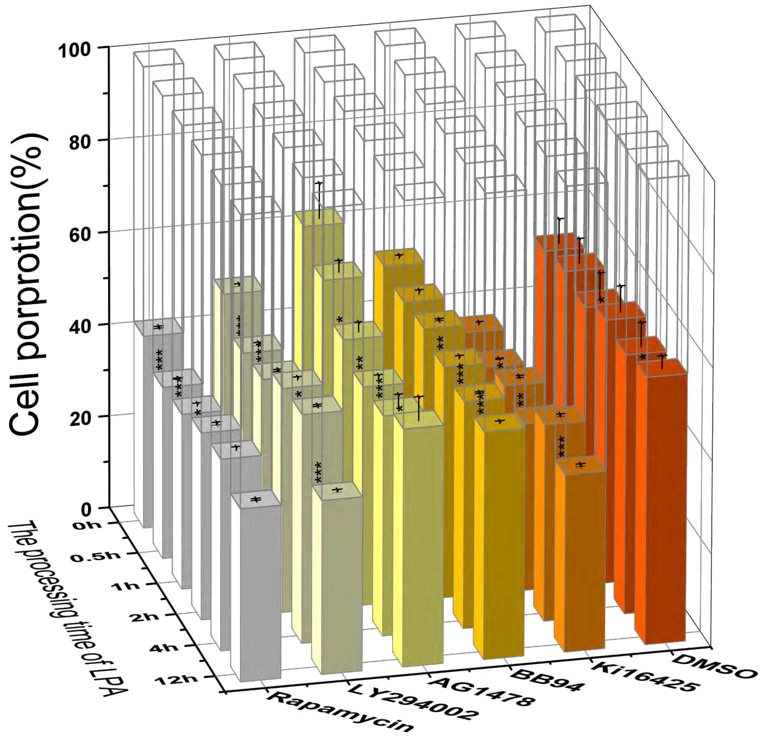
Cell porprotion(%)
100
80
60
40
20
0
The processing time of LPA
0h
0.5h
1h
2h
4h
12h
Rapamycin
LY294002
AG1478
BB94
Ki16425
DMSO
